# Polarimetric observables for the enhanced visualization of plant diseases

**DOI:** 10.1038/s41598-022-19088-6

**Published:** 2022-08-30

**Authors:** Carla Rodríguez, Enrique Garcia-Caurel, Teresa Garnatje, Mireia Serra i Ribas, Jordi Luque, Juan Campos, Angel Lizana

**Affiliations:** 1 Optics Group, Physics Department, Universitat Autònoma de Barcelona, 08193 Bellaterra, Spain; 2 LPICM, CNRS, Ecole Polytechnique, Institut Polytechnique de Paris, 91120 Palaiseau, France; 3 Botanical Institute of Barcelona (IBB, CSIC-Ajuntament de Barcelona), 08038 Barcelona, Spain; 4 Institute of Agrifood Research and Technology (IRTA), 08348 Cabrils, Spain

**Keywords:** Biophysics, Plant sciences, Optics and photonics, Physics

## Abstract

This paper highlights the potential of using polarimetric methods for the inspection of plant diseased tissues. We show how depolarizing observables are a suitable tool for the accurate discrimination between healthy and diseased tissues due to the pathogen infection of plant samples. The analysis is conducted on a set of different plant specimens showing various disease symptoms and infection stages. By means of a complete image Mueller polarimeter, we measure the experimental Mueller matrices of the samples, from which we calculate a set of metrics analyzing the depolarization content of the inspected leaves. From calculated metrics, we demonstrate, in a qualitative and quantitative way, how depolarizing information of vegetal tissues leads to the enhancement of image contrast between healthy and diseased tissues, as well as to the revelation of wounded regions which cannot be detected by means of regular visual inspections. Moreover, we also propose a pseudo-colored image method, based on the depolarizing metrics, capable to further enhance the visual image contrast between healthy and diseased regions in plants. The ability of proposed methods to characterize plant diseases (even at early stages of infection) may be of interest for preventing yield losses due to different plant pathogens.

## Introduction

Polarimetric instrumentation and methods are of interest in a wide range of applications, as for instance, in astronomy^1^, atmospheric pollution studies^2,3^, security and remote sensing^4^, materials characterization^5^, biomedicine^6^, etc. In the case of applications in biophotonics, polarimetric methods have proved to be very useful tools to enhance the image contrast of some organic structures, and/or providing information of certain structures invisible by using regular (non-polarimetric) images. This situation is useful, for instance, for the early detection of some diseases, such as breast cancer^7,8^, colon cancer^9^, skin cancer^10^, or brain cancer^11^, among others.

The above-stated use of polarimetric methods for the study of animal^12^ or even human^8–10^ tissues is a well-established field of work^13^ and nowadays continues in constant development. However, the application of polarimetric methods for the study of plant diseases is less common and, in the last decade, there is a growing interest of exploring more complex (and rich in terms of information) polarimetric solutions for applications in plant science. Historically, one of the most widely used polarization-based optical instruments for studying plant structures is the polarimetric microscope^14^. This instrument allows clear observation of vegetal cells, as centrosome-nuclear complexes in cell division^15^ or cell suspension culture of plant specimens, as in *Picea glauca*^16^. Note that polarimetric features exploited by polarimetric microscopes are dichroism and birefringence, when they are present in samples. For instance, in the case of vegetal structures, dichroism measurements reveal the concentration and spatial organization of some plant organelles such as pigment–protein complexes in plant thylakoid membranes^17^, chloroplasts^18^, or quantasomes^19^. Moreover, the birefringence signature of some structures and macromolecules (for instance, cellulose), allows to study the cell wall composition^20^, the trichomes structure^21^ or stomata^22^.

In addition to dichroism and birefringence, depolarization is third polarimetric channel which provide valuable information, although it has been underused in plant characterization. Depolarization is a statistical concept originated by incoherent (temporal or spatial) addition of different light polarizations at the level of the detector, and it can be understood as the degree of polarization disorder (randomness) introduced by a given structure to an input polarization. A suitable tool for studying depolarizing information are Mueller polarimeters^23,24^, that commonly performs macroscopic analysis of samples. Recent works^4,25,26^ have shown and discussed the advantages of the depolarization-related observables to enhance image contrast between different plant structures of interest, as midrib, secondary veins, stomata and raphides. This is because their constituent units (cellulose, pectin, water content, among others) present different polarimetric features (retardance or dichroism) and/or different spatial organization, these situations inducing depolarization at macroscopic scale observation. Importantly, depolarization methods have proved to be a very interesting tool for the inspection of plant tissues, independently or in combination to other optical instrumentation, as spectroscopic instrumentation, phase or fluorescent microscopy, among others^25,27^.

A popular figure or metric to study the depolarizing response of plant structures has been the so-called degree of polarization (DoP)^26,28^, which measures the global change in the polarization degree of a radiation beam after interacting with a given material media, in our case, a plant tissue. This metric has been used, for instance, to estimate the chlorophyll content^29^, the plant stress^30^ and for vegetation classification purposes^31^. The description of the depolarization provided by the DoP, can be refined with the use of the so-called depolarization metric sets as it has been discussed in recent works^25,26^. A depolarization space is a combination of at least three depolarization-related metrics which are derived from the Mueller matrix, to enhance the image contrast in plant structures^4,25,26,32^. This is the case of the so-called Indices of Polarimetric Purity (IPPs)^33,34^, that are connected with the type of polarimetric randomness that a system induces to incident light (they give information of the depolarization anisotropy of the system, i.e., dependence of the input polarization with the resultant depolarization response).

When it comes to plant pathogens, virial infections in plant specimens can induce, among others, a decrease in photosynthesis through decreases in chlorophyll efficiency and the disruption of cellular processes. In turn, the damaged cellular metabolism induces the development of abnormal substances which are injurious to the functions of the plant. Furthermore, plant diseases caused by fungi infections are generally described by the pathogen consuming cells or secreting toxins. These may lead to plant tissues containing a mix of both necrotic (dead) and healthy tissues intermingled with the fungal mycelium and may induce to microscopic structural changes^35^. Those biological modifications in plants caused by virus and fungi may be the origin of depolarization differences between healthy and pathological tissues in plants, but more study must be developed in this research line to connect microscopic biological changes with macroscopic depolarization measures. In the present paper we discuss for the first time, the use of polarimetry and in particular the use of depolarization-related metrics for the visualization and characterization of plant diseases. In particular we show how depolarizing metrics are suitable for the discrimination between healthy tissues and different type and stages of plant infections. The depolarization set of metrics chosen for the present study is the IPPs set because, as stated before, it provides three suitable metrics for plant visualization^25,26^. In addition to IPPs, we include in the study, for completeness, a second set of depolarizing observables: the so-called Components of Purity (CPs)^36^. The selection of these two polarimetric spaces is not arbitrary. Whereas the IPPs describe the capability of depolarizing samples to introduce polarimetric randomness to incident light, the CPs provide information of the polarimetric characteristics in samples inducing depolarization (diattenuation, polarizance and retardance). Importantly, these two spaces result in complementary analytical tools, and their combined use completely describes the polarimetric behavior of depolarizing samples^37^. We further demonstrate the suitability, from a quantitative and qualitative point of view, of such depolarizing spaces for plant diseases visualization enhancement. The analysis is conducted on a set of different botanical specimens showing different disease symptoms and injury stages due to the particular infection of different pathogen agents. Moreover, the potential of the method is strengthened by applying a pseudo-colored approach that helps to magnify the visual contrast between healthy and diseased tissues, or between different stages of the disease. We believe that the use of polarimetric methods, which are non-invasive and non-contact, for the early detection of plant diseases are of interest because they may contribute to prevent large product (and economical) losses in crops^38,39^.

## Results

**Figure 1:**
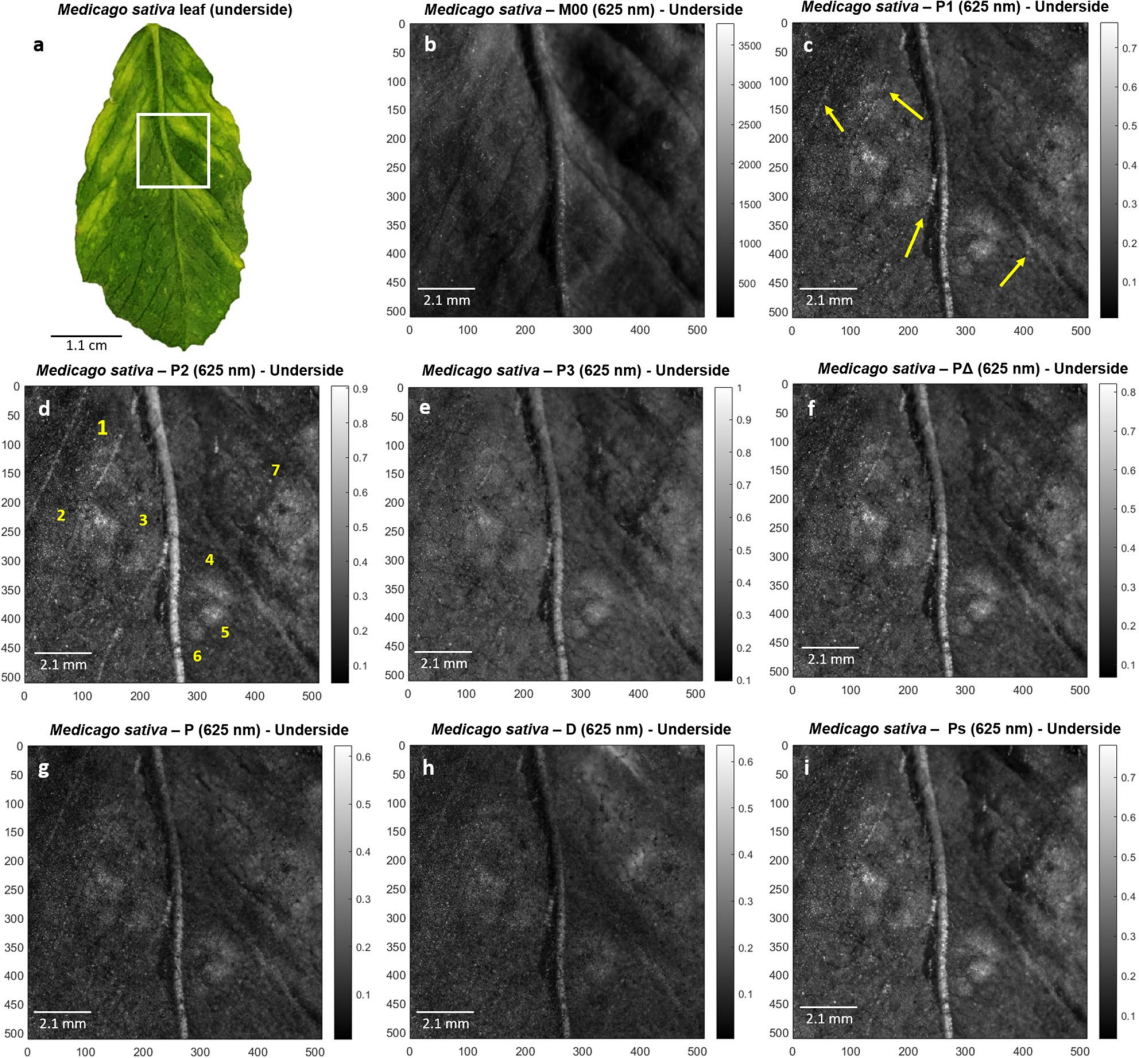
Polarimetric images of *Medicago sativa* leaf used in this study. (**a**) Picture of the underside part of the *Medicago sativa* leaf. White square denotes for selected region of interest (ROI) analyzed in remaining images, (**b**) regular intensity image (M_00_) of the *M. sativa* underside ROI and its corresponding polarimetric observables (**c**) *P*_1_, (**d**) *P*_2_, (**e**) *P*_3_, (**f**) *P*_*Δ*_, (**g**) *P*, (**h**) *D* and (**i**) *P*_*S*_ for visual comparison. All images correspond to 625 nm illumination wavelength measurements performed at scattering set-up configuration. Yellow arrows correspond to the enhanced vascular structures within the sample, whereas numeric labels (from 1 to 7) indicate the number of chlorotic spots unraveled by means of polarimetric observables.

**Figure 2:**
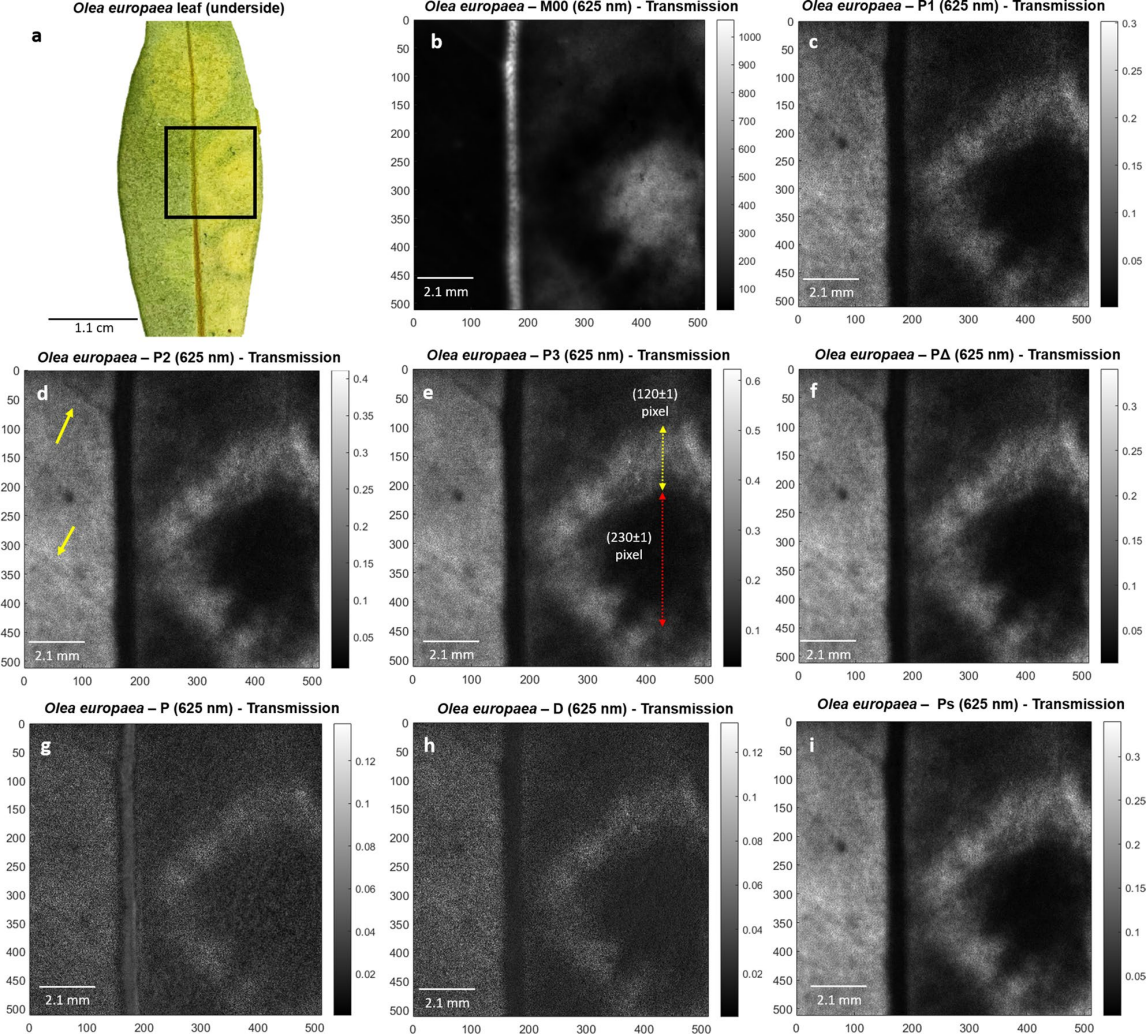
Polarimetric images of *Olea europaea* leaf used in this study. (**a**) Picture of the underside part of *O. europaea* leaf. Black square denotes for selected region of interest (ROI) analyzed in remaining images, (**b**) regular intensity image (M_00_) of the *O. europaea* transmission ROI and its corresponding polarimetric observables (**c**) *P*_1_, (**d**) *P*_2_, (**e**) *P*_3_, (**f**) *P*_*Δ*_, (**g**) *P*, (**h**) *D* and (**i**) *P*_*S*_ for visual comparison. All images correspond to 625 nm illumination wavelength measurements performed at transmission set-up configuration. Red and yellow dotted lines correspond to diameter and width measurements for chlorotic spot and necrotic ring, respectively. Yellow arrows indicate the unveiled vascular structures.

The results presented in this work are related to the study of two leaf specimens suffering different infections: (1) a leaf from a specimen of *Medicago sativa* (alfalfa), which was found infected with *alfalfa mosaic virus* (AMV); and (2) a leaf from a specimen of *Olea europaea* (olive), infected with the fungus *Venturia oleaginea* (olive leaf spot). We have bounded the study in these two representative samples, for the impact and utility of these specimens in humans, but the main conclusions of this works can be extrapolated to other specimens and infectious agents. In particular, we tested the methods in a set of 73 leaves corresponding to 18 plant diseased species (complete list presented in Supplementary Table S1) and the obtained results agree. In the particular case of study, the *M. sativa* and the *O. europaea*, both leaves showed the characteristic lesions of their respective diseases. *Medicago sativa* showed chlorotic areas surrounding the affected vascular structure (see Fig. 1a and Supplementary Fig. S1a). In turn, *O. europaea* showed alternating necrotic and chlorotic ring-like lesions surrounding a chlorotic spot (see Fig. 2a and Supplementary Fig. S2a). A description of the plants used on the present study is included in the “Methods” section.

The potential of polarimetric observables for the characterization of the above-mentioned diseases was studied. The polarimetric and standard intensity images of the *M. sativa* and *O. europaea* leaves were taken by means of a complete image Mueller polarimeter working at three different illumination wavelengths (625 nm, 530 nm and 470 nm) in scattering and transmission configurations. In the context of Mueller matrix-derived images, we refer to the standard intensity (i.e., the M_00_ coefficient) as the non-polarized intensity image due to the fact that it may be interpreted as the image that would have been taken if the sample was illuminated with natural (or unpolarized) light^36^. A representative example of standard intensity images of samples can be seen in Figs. 1b and 2b. In addition, the detailed description of the polarimeter used in this study can be found in “Methods” section. The results presented here correspond to the underside part of the chlorotic *M. sativa* and *O. europaea* leaves measured in scattering and transmission configuration, respectively, at a wavelength of 625 nm because they represent the most relevant and interesting findings in terms of image enhancement using the polarimetric approach. With regards to the illumination channel selection, 625 nm wavelength light penetrates more deeply into the sample than shorter wavelengths (530 nm or 470 nm in our case). In this way, in the case of *O. europaea* leaf, light is capable to reach the opposite surface of the leaf, providing information about the diseased tissues at all depth levels. Similar behavior occurs for *M. sativa* leaf, where 625 nm illumination wavelength measurements provide a more accurate description of the lesions. Complementary measurement configurations and their respective obtained polarimetric images are presented in Supplementary Figs. S1 and S2.

For the optimal presentation and interpretation of results, we perform a qualitative analysis by comparing the acquired standard intensity images with the polarimetric observables described in “Methods” section. For consistency, the analysis is complemented with quantitative study regarding the polarimetric behavior on different structures of healthy and diseased tissues of samples. We also present a pseudo-colored approach image technique that allows a better visualization of certain healthy and diseased plant structures.

### Polarimetric analysis of chlorosis and necrosis

By taking advantage of the different depolarizing behavior of biological structures in samples^40^, we calculated different Mueller matrix-derived polarimetric observables images and we compared them with standard intensity images, for the two specimen leaves analyzed. The selected observables for the analysis of plant samples, which are described in the “Methods” section, provide a complete description of the enpolarizing properties of samples^37^. In particular, we use the indices of polarimetric purity—*P*_1_, *P*_2_, *P*_3_ -, the components of purity—*P*, *D*, *P*_*S*_—and the depolarization index *P*_*Δ*_, for the polarimetric sample description. The obtained results show that the depolarization metrics *P*_1_, *P*_2_, *P*_3_, *P*_*Δ*_ and *P*_*S*_ clearly manifest an overall enhancement of image contrast and they help to unveil wounded zones or vascular structures that are invisible by using standard non-polarized images. In the following we present two illustrative results. The standard non-polarized transmission images for the *M. sativa* and *O. europaea* leaves are shown in Figs. 1b and 2b, respectively. These images were obtained from certain regions of interest (ROIs) in the leaves, indicated with white and black squares in Figs. 1a and 2a, respectively. For comparison purposes, we provide the polarimetric images corresponding to the analyzed polarimetric observables, identifying the ones that provide the largest image enhancement, i.e., the *P*_2_ channel for the *M. sativa* case (Fig. 1d) and the *P*_3_ channel for the *O. europaea* case (Fig. 2e).

Importantly, thanks to the contrast enhancement of polarimetric observables in the visualization of the *O. europaea* image, we can clearly distinguish the necrotic ring and chlorotic spot edges visible in different polarimetric channels (Fig. 2c–i), the necrotic ring being invisible by using non-polarized light intensity images (Fig. 2b). A thorough discussion of the visualization of disease symptoms by using polarimetric means as well as the current sample chlorotic spot and necrotic ring characteristics (diameter and width, red and yellow dotted line in Fig. 2e, respectively) is provided in the “Discussion” section.

**Figure 3:**
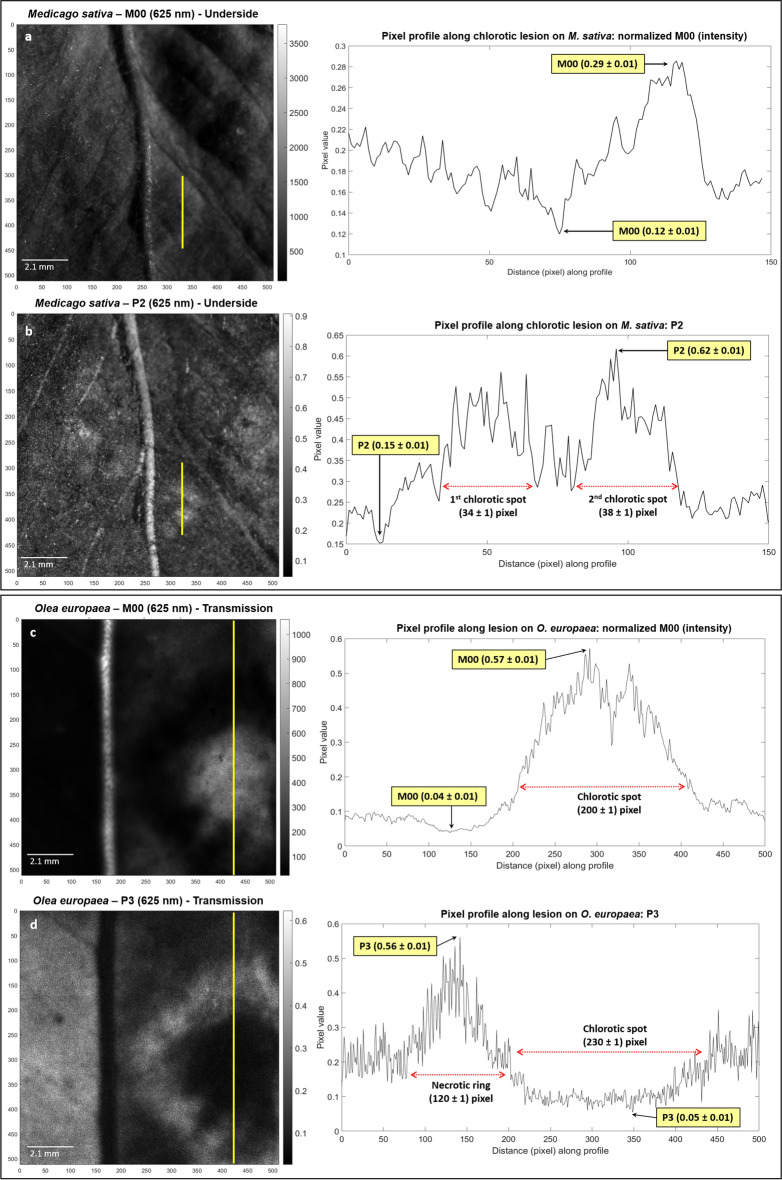
Pixel profile comparison for *Medicago sativa* and *Olea europaea*. (**a**) Intensity image at 625 nm of the underside part of *M. sativa* sample and its corresponding pixel profile, (**b**) polarimetric purity index *P*_2_ and its corresponding pixel profile, (**c**) intensity image at 625 nm of the underside part of *O. europaea* sample and the corresponding healthy-necrotic-chlorotic transition pixel profile, and (**d**) polarimetric purity index *P*_3_ and its corresponding pixel profile. The vertical yellow lines on polarimetric images indicate the plotted pixel profile segments. The squared numeric labels for M_00_, *P*_2_ and *P*_3_ indicate their respective highest and lowest pixel values within the inspected pixel regions. Red-dotted horizontal lines on plots indicate the diameter of the two chlorotic spots of *M. sativa* and width measurements for chlorotic spot and necrotic ring of *O. europaea*.

As a complement to the above-presented images, we quantify the potential of depolarizing metrics to characterize plant pathologies. To do so, we consider the values taken along different cross-sections in the images of the observables shown in Figs. 1 and 2. The particular cross-sections analyzed, highlighted in yellow lines, together with the corresponding values are shown in Fig. 3. Figure 3a and b present a comparison between the values of the classical unpolarized light intensity transmission (M_00_) metric with the depolarization metrics *P*_2_. The values of the cross-section displayed correspond to the transition between a healthy and a chlorotic zone in the *M. sativa* specimen leaf. The difference between the chlorotic and the heathy areas is hardly visible using the classical M_00_ observable, while it becomes quite apparent in the *P*_2_ metric image. Healthy areas show *P*_2_ values around 0.20 ± 0.01 while chlorotic areas show characteristic values around 0.50 ± 0.01. Analogously, Fig. 3c and d show a comparison between the values of the M_00_ metric and the *P*_3_ metric for the *O. europaea* case. The selected cross-section displays the transition between healthy, necrotic, and chlorotic regions. As in the previous example, the classical M_00_ observable (Fig. 3c) is not sensitive to all the features in the plant, for instance it does not delimitate the necrotic ring, while the *P*_3_ observable (Fig. 3d) clearly discriminates between healthy, necrotic and chlorotic. For instance, the value of *P*_3_ for healthy, necrotic and chlorotic areas is around 0.20 ± 0.01, 0.45 ± 0.01 and 0.10 ± 0.01 respectively. Such selectivity results in a clear enhancement of the contrast of *P*_3_ images when compared to M_00_ images. In the example shown in Fig. 3d the necrotic area is clearly visible and appears as a “ring” area delimited between the chlorotic spot and the healthy tissue. The chlorotic area appears as dark spot, whereas the healthy area appears in grey. In contrast, M_00_ observable shows the chlorotic area as a bright spot with values close to 0.50 ± 0.01, the necrotic ring in dark, typical values of 0.05 ± 0.01, and the healthy area in dark grey with typical values of 0.90 ± 0.01. The difference between healthy and necrotic areas is less net in a M_00_ image than in a *P*_3_ one.

**Figure 4:**
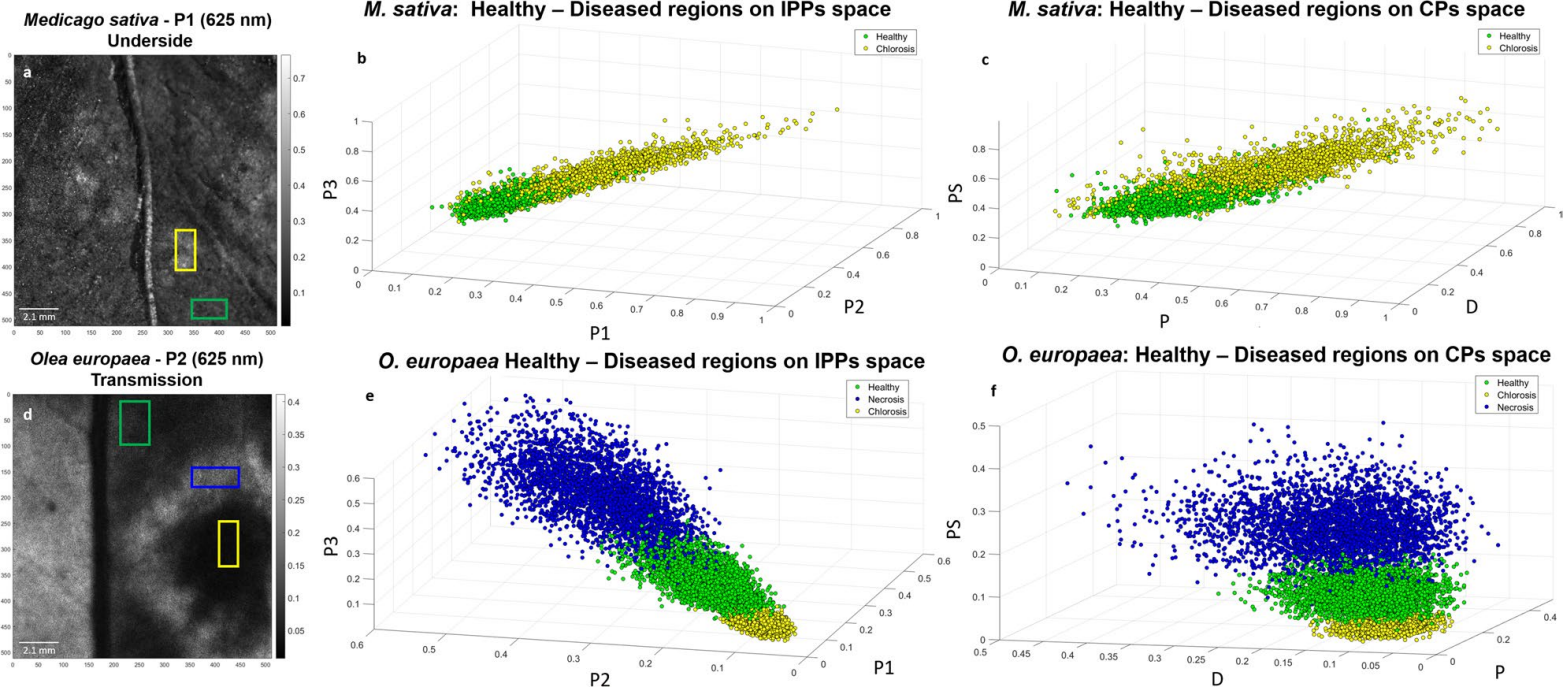
Scatter data plots of healthy and diseased tissue regions of *Medicago sativa* and *Olea europaea*. (**a**) Visual indicative for healthy (green) and chlorotic (yellow) tissue selected region of interest (ROI) for *M. sativa*, (**b**) corresponding IPPs space (*P*_1_, *P*_2_, *P*_3_) for healthy and chlorotic data clouds representation, (**c**) components of purity (*P*, *D*, *P*_*S*_) space for healthy and chlorotic data clouds representation, (**d**) visual indicative for selected healthy (green), chlorotic (yellow) and necrotic (dark blue) tissue ROIs for *O. europaea*, (**e**) corresponding IPPs space (*P*_1_, *P*_2_, *P*_3_) for healthy, chlorotic and necrotic data clouds representation and (**f**) components of purity (*P*, *D*, *P*_*S*_) space for healthy, chlorotic and necrotic data clouds representation.

We now further analyze the potential of those observables to discriminate between different typologies (healthy or diseased tissues) of plant structures. To do so, we represent the measured data on different polarimetric spaces^41^, this leading to a very intuitive visualization of data, and also providing quantitative information of the structures (or tissue types) that may be present in the images of the probed samples. Moreover, the data in each ROI is collected and grouped in what we call a data-cloud which can be used for ulterior statistical data treatment or for graphical representation. For instance, data from a homogeneous region should show close values with little variance, in contrast to data from a heterogeneous region, which should show different values with extended deviation from a mean value. When represented in a graph, data from a homogeneous region may group within a well-defined region, whereas data from an heterogenous region may tend to spread over an irregular volume. Therefore, a graphical representation of data-clouds is a good tool to see at a glimpse the variability and the average values of such data. When a given area in a sample is represented according to various observables, such for instance M_00_, IPPs, or CPs, then, data clouds corresponding each to a given observable, can be defined. Figure 4 presents the data clouds from selected ROIs (marked in Fig. 4a and d as colored rectangles) of healthy (green), chlorotic (yellow) and necrotic (dark blue) tissue regions of *M. sativa* (first row in Fig. 4) and *O. europaea* (second row in Fig. 4) leaves. The size of the corresponding regions of interest were selected depending on the tissue availability of sample to perform a homogeneous selection of healthy and diseased tissue. In particular, healthy and chlorotic tissue regions on *M. sativa* correspond to a total of 2800 pixels (40 × 70 and 70 × 40 pixels, respectively.) In counterpart, healthy, necrotic and chlorotic ROIs for *O. europaea* are of 90 × 40 (3600 pixels), 30 × 110 (3300 pixels) and 70 × 40 (2800 pixels), respectively. The data clouds are represented in the IPPs space for the *M. sativa* (Fig. 4b) and *O. europaea* (Fig. 4e), as well in the CPs space (Fig. 4c,f, respectively). Whereas the *M. sativa* presents two differentiated structures (healthy and chlorotic), the *O. europaea* presents three differentiated structures (healthy, necrotic and chlorotic). We see as in both plant specimens, and for the two studied polarimetric spaces, the different leaf structures (healthy, necrotic and chlorotic) are clearly differentiated and located in different spatial positions without data mixing (the different type of data -different colored dots- practically do not overlap in any region of the spaces). This trend was also observed when selecting other affected regions and the selected examples in Fig. 4 are representative cases that illustrate the discriminatory potential of polarimetric observables for symptom detection and description.

### Pseudo-colored approach

In this section, we want to go one step further in plant pathology imaging based on polarimetric observables by using the discussed polarimetric spaces into a pseudo-coloration image method^42–44^. The main idea consists of building a “polarimetric triplet” by selecting, between all the used polarimetric observables, the three of them leading to larger image contrast between healthy and diseased tissues within the inspected sample. Afterwards, each one of the selected polarimetric images of the triplet is associated with a primary color (red, green and blue, respectively), and they are properly combined to build pseudo-colored images providing visual contrast between plant structures of interest. Detailed description of the pseudo-colored approach proposed in this study can be found in Supplementary Sect. 3.3.

**Figure 5:**
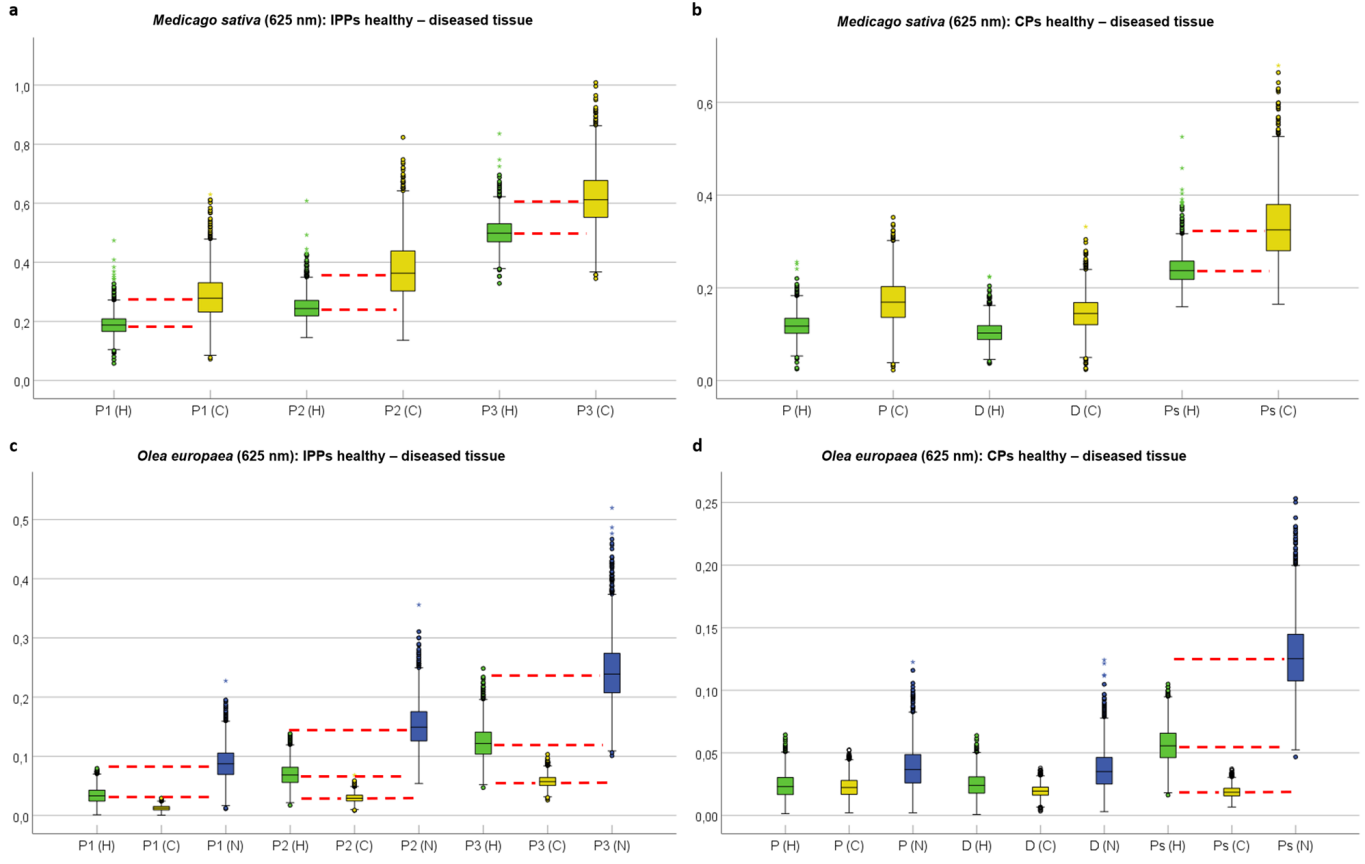
Boxplot charts for healthy and diseased regions for *Medicago sativa* and *Olea europaea*. (**a**) Indices of polarimetric purity (*P*_1_, *P*_2_, *P*_3_) boxplot for healthy and chlorotic locations on *M. sativa*, (**b**) Components of purity (*P*, *D*, *P*_*S*_) Boxplot for healthy and chlorotic locations on *M. sativa*, (**c**) Indices of polarimetric purity (*P*_1_, *P*_2_, *P*_3_) Boxplot for healthy, necrotic and chlorotic locations on *O. europaea* and (**d**) Purity components (*P*, *D*, *P*_*S*_) Boxplot for healthy, necrotic and chlorotic locations on *O. europaea.* The corresponding healthy, chlorotic and necrotic data distributions are labeled and colored as H (green), C (yellow) and N (dark blue), respectively. Red-dashed lines indicate the locations of median values and illustrate they do not fit within the boxes of different tissue conditions (healthy, chlorotic or necrotic), allowing discrimination. Circles and stars correspond to mild and extreme outlier values, respectively.

To achieve an optimal use of the pseudo-color approach, the three observables used to represent the RGB base must be the ones that better discriminate between healthy and wounded areas. The discrimination ability of a given set of observables can be quantified by measuring the differences of these observables when applied to image healthy and wounded areas. To estimate these differences, we performed a Boxplot analysis^45,46^ for the regions of interest (ROIs) shown in Fig. 4. In Fig. 5 we show the split Boxplot analysis of the Indices of Polarimetric Purity (*P*_1_, *P*_2_, *P*_3_) and the Components of Purity (*P*, *D*, *P*_*S*_) for healthy, chlorotic and necrotic tissue regions of *M. sativa* (Fig. 5a,b) and *O. europaea* (Fig. 5c,d).

We can see in Fig. 5 that some observables clearly separate the studied necrotic, chlorotic and healthy tissues. To help readers to evaluate such differences, the median values are highlighted with dotted red lines in the case of largest differences. In the case of the *M. sativa,* which presents chlorotic and healthy tissues, they are clearly separated by all the IPPs (*P*_1_, *P*_2_, *P*_3_) metrics (Fig. 5a) as well as by the sphericity degree observable, *P*_*S*_ (Fig. 5b). In the case of the *O. europaea*, which presents chlorotic, necrotic and healthy tissues, we see how the same observables (IPPs and sphericity degree *P*_*S*_) are those leading to the larger distances between different tissues (Fig. 5c,d). Under these results, the IPPs as well as the sphericity degree, *P*_*S*_, are considered to be good candidates to implement the pseudo-colored images. Moreover, Boxplot unveils the amount of outlier values (mild and extreme, illustrated as small circles and stars in Fig. 5, respectively) of each data distribution so that the homogeneity of the selected tissue region can be quantified. The current distributions of outliers range from 0.44 to 3.67%. Note that the low percentage of data outliers ensures the homogeneous selection of the tissue conditions (healthy, necrotic or chlorotic). The complete description of outlier values for each analyzed tissue region can be found in Supplementary Table S2.

In addition to the above-presented Boxplot analysis, we computed, for each observable in Fig. 5, the distance between medians in yellow and green boxes (chlorotic and healthy, respectively) for the *M. sativa* case (Fig. 5a,b); and between medians in yellow and green boxes (chlorotic and healthy, respectively) as well as between medians in blue and green boxes (necrotic and healthy, respectively) for the *O. europaea* case (Fig. 5c,d). The values resulting from such comparison are shown in Table 1. The largest values in Table 1 are highlighted in bold. The median values of each polarimetric observable for selected healthy and diseased regions on both plant species are shown in Supplementary Table S3.

**Table 1:** Polarimetric observables median value difference and propagated errors for healthy-diseased tissue.

	*P*_1_	*P*_2_	*P*_3_	*P*	*D*	*P*_*S*_
***Medicago sativa***						
Chlorotic—Median diff	0.091 ± 0.085	**0.119 ± 0.104**	0.113 ± 0.106	0.051 ± 0.055	0.042 ± 0.043	0.088 ± 0.081
***Olea europaea***						
Chlorotic—Median diff	0.020 ± 0.014	0.039 ± 0.020	**0.064 ± 0.029**	0.001 ± 0.013	0.004 ± 0.011	0.037 ± 0.015
Necrotic—Median diff	0.054 ± 0.031	0.080 ± 0.041	**0.117 ± 0.059**	0.013 ± 0.019	0.011 ± 0.019	0.069 ± 0.032

The corresponding three largest median difference values are highlighted in bold.

By considering the largest differences between means according to Table 1, we selected two triplets of polarimetric observables for the pseudo-colored images construction: one mixing observables of the IPPs and CPs spaces (*P*_2_, *P*_3_, *P*_*S*_) and another based on the IPPs by themselves (*P*_1_, *P*_2_, *P*_3_). At this stage, for each depolarizing observable selected *P*_*i*_ (where *i* = 1, 2, 3, *S*) we set different threshold values, which were derived from the Boxplot analysis (see Supplementary Table S5). These thresholds allowed us to numerically discriminate between different tissue conditions: healthy/chlorotic for *M. sativa* and healthy/chlorotic/necrotic, for *O. europaea*, and then, each separated condition is assigned to a primary color (red, green and blue, for chlorotic, healthy, and necrotic tissues, respectively). This process is illustrated by considering particular examples. In the case of the *M. sativa*, and for a particular observable selected *P*_*i*_, a first binary image (black–red) is constructed according to pixel values above/below a certain threshold, this image carrying the chlorotic information of the plant. Then, a second binary image (black–green) is similarly constructed carrying the healthy information of the plant. The same procedure is conducted for the for *O. europaea,* but now, as this specimen presents an extra condition (necrosis), three binary-images are obtained: (1) for the chlorotic content (black–red), (2) for the healthy content (black–green), and (3) for necrotic content (black–blue). Finally, for each studied metric (*P*_1_, *P*_2_, *P*_3_, *P*_*S*_), a final pseudo-colored image is obtained by adding all the contributions as following,

1 $$P_{i,approach} (x,y) = P_{i,Red - Chlorotic} (x,y) + P_{i,Green - Healthy} (x,y) + P_{i,Blue - Necrotic} (x,y)$$

where the sub-index *i* denotes for the particular depolarizing observable (i.e., *i* = 1, 2, 3,* S*). Note that in the case of the *M. sativa* we can consider *P*_*i, Blue-Necrotic*_ = 0 because there is not necrotic content. Importantly, each term in Eq. (1) provides a binary-colored image that has been filtered according to the threshold criteria above-explained. Finally, the content of a full polarimetric triplet, [*P*_1_, *P*_2_, *P*_3_] or [*P*_2_, *P*_3_, *P*_*S*_], is put together by constructing a linear combination of the pseudo-colored observables implemented according to Eq. (1), so generalized final pseudo-colored images, valid for both *M. sativa* and *O. europaea*, are obtained as:

2 $${\text{Pseudo}}\# 1(x,y) = P_{2,approach} (x,y) + P_{3,approach} (x,y) + P_{S,approach} (x,y),$$

3 $${\text{Pseudo}}\# 2(x,y) = P_{1,approach} (x,y) + P_{2,approach} (x,y) + P_{3,approach} (x,y).$$

As shown in Eqs. (2) and (3), we built two general pseudo-colored functions (corresponding to each of the two selected observables triplets) labeled as #1 and #2. Note that both Eqs. (2) and (3) can be applied for the two plant specimens analyzed: The *M. sativa* and the *O. europaea* cases. Note as well that the corresponding weights of each term in Eqs. (2) and (3) were chosen, for simplicity, as the unit, this giving the same weight to all the observables in the triplet, but other weights could be selected to enhance plant structures visualization. In fact, the low values of the selected polarimetric observables in the case of the *O. europaea* (see Supplementary Table S3) lead to a darkened final image. In such a case, to visually improve the pseudo-coloration approach, the weights were pondered by a factor 2 so that the resulting image turned brighter. A more detailed description of the method proposed to build pseudo-colored functions is provided in Supplementary Sect. 3.3.

**Figure 6:**
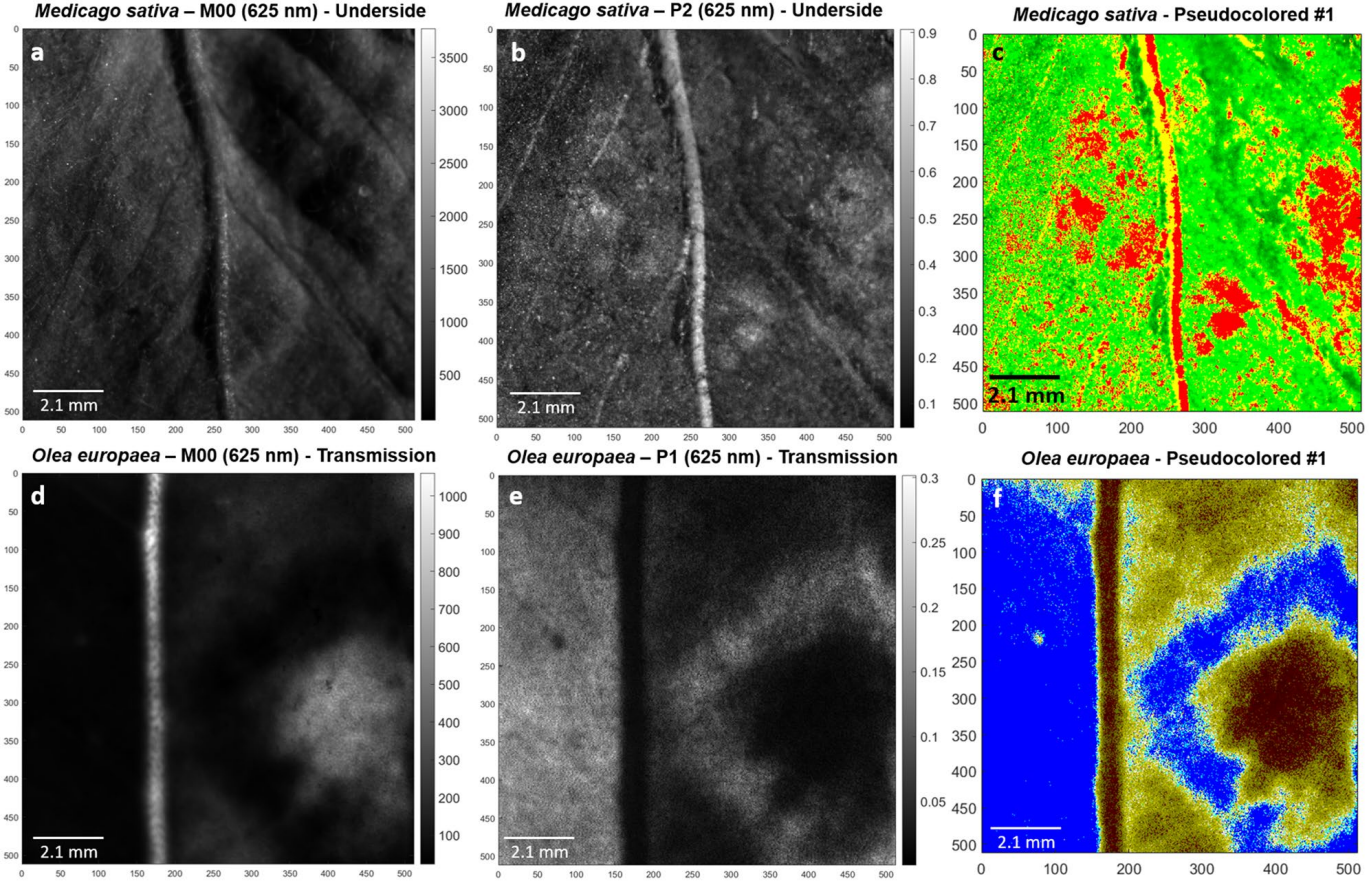
Visual comparison of *Medicago sativa* leaf: (**a**) 625 nm intensity image (M_00_), (**b**) polarimetric purity index *P*_2_, (**c**) processed image by means of #1 pseudo-coloration; Visual comparison of *Olea europaea* leaf: (**d**) 625 nm intensity image (M_00_), (**e**) polarimetric purity index *P*_1_, (**f**) processed image by means of #1 pseudo-coloration.

Figure 6 shows three images that illustrate the effect of pseudo-coloration to enhance the contrast of polarimetric images as well as the interest of this technique to discriminate between different tissues. The figure shows images of classical M_00_, the representative polarimetric observable *P*_2_ and the corresponding pseudo-colored image for *M. sativa*, and *P*_1_ for *O. europaea*. In particular the first row in Fig. 6 presents the classical non-polarizer transmission (M_00_, Fig. 6a), the polarimetric purity index *P*_2_ (Fig. 6b), and pseudo-coloration resulting from Eq. (2), labeled as #1 (Fig. 6c), for *M. sativa* leaf sample. The second row in Fig. 6 presents the intensity (Fig. 6d), the polarimetric purity index *P*_1_ (Fig. 6e), and pseudo-coloration resulting from Eq. (2), labeled as #1 (Fig. 6f), for *O. europaea* leaf. We found that the pseudo-colored resulting images for purity-mixed space and isolated purity space both shown very similar results. To not to present redundant information, pseudo-colored images regarding isolated Indices of Polarimetric Purity [Eq. (3)] for *M. sativa* and *O. europaea*, are presented in Supplementary Fig. S4d and h, respectively. The images resulting from polarimetric based pseudo-colored processing are excellent in terms of enhanced visual discrimination of healthy/diseased tissues. Detailed analysis of enhanced structures is provided in “Discussion” section.

## Discussion

The present work highlights the suitability of using polarimetric observables, in particular, the Indices of Polarimetric Purity (IPPs) and the Components of Purity (*P*, *D* and *P*_*S*_), for the inspection of plant disease symptoms. In addition, we show that the implementation of a pseudo-coloration image processing method, which is based on the above-mentioned polarimetric observables, is a useful tool to enhance the image contrast between different tissue natures (healthy, necrotic and chlorotic) of botanical samples. Although the potential of depolarizing metrics to characterize different plant pathologies was observed in 18 different species affected by different infection agents, we focus our attention in two specific plant species because of their relevance in agricultural production: alfalfa (*M. sativa*) and olive (*O. europaea*). The complete list of the specimens used in this study is presented in Supplementary Table S1.

Normal levels of chlorophyll in plant cells are the cause of the characteristic green color of leaves and allow the natural performance of photosynthetic processes, i.e., the synthesis of organic substances like carbohydrates, from the sources of inorganic prime matter and water from soil, CO_2_ from the atmosphere, and light^35^. Chlorophyll deficiency in plant foliage triggers the appearance of chlorosis: the lack of green pigments, which leads to the yellowing of tissues, thus hampering the production of nutritional substances. The chlorotic symptoms can appear due to different biotic and abiotic causes, e.g., iron-deficient or alkaline soils (high pH levels), and plant pathogens^35^, such as the alfalfa pathosystem (AMV) studied here. With regards to the necrotic symptomatology caused by a pathogen infection, it is characterized by the premature death of affected plant cells and the darkening of tissues. The release of pathogen toxins into the plant cell or, additionally, the release of residual components from surrounding dead cells into the intracellular space typically induces the necrotic lesion appearance. In this study we selected the case of necrosis in leaves of *O. europaea* as an illustrating example.

All plant samples were inspected at different wavelengths (625 nm, 530 nm and 470 nm) and measuring configurations (scattering–measuring both the beam and the underside part of the samples, and transmission). However, to not to extent the content of the manuscript, we only discuss here the cases providing the most interesting results in terms of disease symptom visualization. In particular, we consider *M. sativa* leaves measurements at 625 nm in scattering configuration from the underside, and the *O. europaea* leaves measured in transmission configuration from the outside. Measurements with additional orientations of the leaves were performed and the corresponding results are summarized in the Supplementary document.

Although different optical leaf properties may play an important role in leaves spectral response^40^, as for example the spectral signature of a leaf, in the specimens studied in this work we have observed that longer wavelengths penetrate more into samples than shorter wavelengths, as they are less affected by scattering processes than shorter wavelengths. This result agrees with discussion provided in Ref.^47^. In fact, longer wavelengths may carry more information about microstructures present in the bulk of the sample than shorter wavelengths, which are more sensitive to features present in surface in a bulk region near the surface of the leaves. Since chlorosis or necrosis affect the whole bulk of the leaf, a relative long wavelength such as 625 nm provides a more complete picture of the leave than a shorter wavelength and it is for this reason why we chose in the results shown in the main text of the present manuscript. A second reason for our choice, is that chlorophyll has an absorption peak close to 625 nm, and although being weak this particular absorption feature will impact the measured depolarization. In transparent scattering media, photons which contribute to increase the depolarization of light are those who followed a higher number of scattering events before reaching the detector. Those photons are the ones which also show longer paths inside the sample because of the multiple scattering events that they experienced. In absorbing media, photons which account a higher number of scattering events will be preferentially removed from the beam because their paths are longer than those of photons which account a fewer number of scattering events. Therefore, and in general, it can be said that an absorbing media is expected to show less depolarization than analogous transparent media. In the cases discussed here, because of chlorotic and necrotic tissues show reduced chlorophyll concentration, they appear more transparent than healthy ones at 625 nm: reason why depolarization measurements at this particular wavelength may be more sensitive to the presence of chlorophyll (and therefore to the presence of a wounded tissue) than measurements at wavelengths for which chlorophyll does not show any absorption.

In the case of the *M. sativa* leaf (Fig. 1), the polarimetric images; in particular, the ones corresponding to the depolarization-related *P*_1_, *P*_2_, *P*_3_, *P*_*Δ*_ and *P*_*S*_ observables (Fig. 1c–f,i, respectively); show a significant enhancement of image contrast between healthy and diseased regions when compared to the image of the standard M_00_ unpolarized transmitted or scattered light intensity. For instance, up to seven chlorotic spots (labeled from 1 to 7 in Fig. 1d) as well as some vascular structures (yellow arrows in Fig. 1c), which are barely observable by standard intensity images (Fig. 1b), are well visible by using polarimetric means. The improvement is further highlighted after comparing the line cross-sections corresponding to the yellow segments in Fig. 3a (regular intensity image, *M*00 channel) and Fig. 3b (*P*_2_ channel), which are taken within healthy and diseased regions in *M. sativa* sample (diseased spots labeled as 4 and 5 in Fig. 1d). In this sense, considering the maximum and minimum values (peak-to-valley) in the Fig. 3a and b pixel profiles (corresponding to the healthy/diseased tissues regions) we demonstrate a significant increase of the chlorotic symptom visibility. For practical reason we quantify visibility with the figure defined by the following expression: $V = \left[ {I_{\max } - I_{\min } } \right]/\left[ {I_{\max } + I_{\min } } \right]$, where I_max_ and I_min_ correspond to the maximum and minimum value of the pixel within the selected segment in the cross-section or the region in the image. In the case discussed here, the visibility features a value of V = (0.41 ± 0.05) for the non-polarized transmission/scattering intensity in Fig. 3a and of V = (0.61 ± 0.03) for the *P*_2_ channel in Fig. 3b.

Moreover, thanks to the visualization and contrast improvement in polarimetric images, it is possible to measure the width of the diseased regions with a pixel precision limited by the density of pixels in the detector. After calibration we found that one pixel of CCD corresponds to approx. 21 µm in sample. Therefore, the size of the chlorotic regions shown in Fig. 1 are estimated to (34 ± 1) pixel, (730 ± 21) µm, and (38 ± 1) pixel, (820 ± 21) µm, for the first and second chlorotic spots, respectively. On the other hand, note that we can physically interpret the depolarizing behavior of healthy/diseased tissues in *M. sativa*, considering that the higher the value of IPPs within a particular tissue region, the lower depolarization induces to incident light^33^. Under this scenario, and according to images in Fig. 1, the chlorotic spots induce fewer depolarizing effects on incident light (high IPPs values) than healthy tissues (low IPPs values) as expected in connection with the previous discussion, because of the reduced absorption of light in the sample due to low levels of chlorophyll.

In the case of the *O. europaea* sample (Fig. 2), it presents a necrotic ring (surrounding a chlorotic spot) hardly visible in non-polarized transmission/scattering images (Fig. 2b). In contrast, polarimetric observables show an increased contrast compared to that of non-polarized transmission/scattering, which provide a more accurate visualization and spatial delimitation of the lesions in the leaf. Moreover, as in the case of *M. sativa* previously discussed, the use of polarimetric observables unveils the presence of vascular structures (see Fig. 2c–i) non-visible in non-polarized transmission/scattering images. Thanks to this visual improvement, the width of the necrotic ring and the diameter of the chlorotic spot are clearly delimited in polarimetric images (yellow and red dotted lines in Fig. 2e, respectively): (120 ± 1) pixel, (2.580 ± 0.021) mm, for the necrotic ring width and (230 ± 1) pixel, (4.940 ± 0.021) mm for the chlorotic spot diameter. The unveiled vascular structures are pointed out with yellow arrows in Fig. 2d. As in the previous case, this visual discrimination between the diseased and healthy regions within the sample can be quantitatively measured analyzing the pixel profile corresponding to the cross-section represented by yellow segments in Fig. 3c (non-polarized transmission/scattering, M00, image) and Fig. 3d (observable *P*_3_). As in the previous case, the cross-sections are selected crossing healthy and wounded areas of the tissue. The chlorotic spot appears as a broad bell-like feature in the non-polarized transmission/scattering cross-section (Fig. 3c) hiding the presence of the necrotic area. In contrast, both the chlorotic spot and the necrotic ring can be distinguished in the cross-section corresponding to the *P*_3_ observable (indicated with a red dashed segment in Fig. 3d). To measure the contrast improvement in polarimetric images compared to non-polarized transmission/scattering ones, we take for instance the visibility value for the necrotic ring which is estimated to V = (0.84 ± 0.03) and V = (0.00 ± 0.03) respectively.

Furthermore, the necrotic ring registers the highest IPPs values (*P*_3_ from 0.15 to 0.56) followed, in descending order, by the healthy leaf lamina (*P*_3_ values ranging from 0.13 to 0.31) and the chlorotic spot (e.g. *P*_3_ from 0.05 to 0.18). This behavior is physically translated as the necrotic ring inducing fewer depolarizing effects on incident light than healthy regions whose response is, in turn, even less depolarizing than the chlorotic spot. Moreover, note that the left side (leaf lamina) of the *O. europaea* sample presents similar values as the necrotic ring for some polarimetric channels (see Fig. 2), although such region does not present necrotic-like tissue condition. This result, which can lead to errors in the physical interpretation of the studied structure, is originated by the curvature of the leaf at that left-region, which leads to out of focus measurements. Under this scenario, the collected polarimetric information of a plant region is affected by out of focus polarimetric information corresponding to other plant regions, all this information being mixed with the actual polarimetric information of the studied structure (the left-side of the leave, in this case). This situation highlights the importance of analyzing well-focused images of plant structures, for the visual inspection of diseased plant samples through polarimetric methods. If due to the non-planar surface of the studied sample a proper image focusing of plant structures of interest cannot be simultaneously ensured by a single image shot, a scanning-based imaging approach is recommended. In this work, we focus on the structures at the right-side of the image, as all structures of interest (healthy and wounded tissues) are in a non-curved and well-focused region of the leaf. To better illustrate the interest of the use of polarimetric images to characterize disease symptoms in vegetal tissues, we chose to collect the pixels from the original image and to represent them as a whole in a data cloud figure. In a data cloud figure, pixels corresponding to different regions should group in separate clouds. Non-overlapping clouds indicate that the related regions are well discriminated. On the contrary, either fully or partially overlapping clouds indicate a poor discrimination of nominally different zones. For the present study, we used three-dimensional data clouds with a selection of variables corresponding to the so-called space of Indices of Polarimetric Purity, (*P*_1_, *P*_2_ and *P*_3_), or the space of the Components of Purity, (*P*, *D* and *P*_*S*_) (see Fig. 4). In the case of the *M. sativa* leaf, the data clouds, corresponding to healthy and chlorotic regions (green and yellow squares in Fig. 4a, respectively), are well-discriminated as these two tissue conditions are clearly spatially separated (i.e., practically no data mixing between tissues with different health condition is produced) when represented in either the: IPPs or the CPs space (Fig. 4b,c, respectively). According to the previous, healthy tissue (green squares) tends to group close to the point (0,0,0) which corresponds to higher depolarization, while chlorotic regions tend to group to areas related with less depolarization. Importantly, a stronger depolarization response occurs when the leaves contain non-organized spatial structures (i.e., neither not aligned nor homogeneously distributed) or an important number of microstructures which efficiently scatter light. In this context, either the biological or the structural changes caused by the chlorotic symptoms of infected regions are translated into a less depolarizing effect on incident light when compared with healthy tissues, thus increasing the sensitivity of depolarizing channels to chlorosis detection. In the case of the *O. europaea* leaf, both depolarizing spaces clearly discriminate between tissue conditions: see how data clouds corresponding to healthy, chlorotic, and necrotic tissue regions in *O. europaea* (green, yellow and blue squares, respectively, in Fig. 4d) are clearly spatially separated in both IPPs and CPs spaces (Fig. 4e,f, respectively). However, in this case, the chlorotic data (yellow squares) are quite close to the point (0,0,0), therefore indicating that, in this case, chlorotic tissue is more depolarizing than healthy tissue (green squares).

The differential depolarizing behavior among the two studied plant species might be related to specific biological characteristics of these species. Regarding the visible chlorotic symptoms that showed a differential polarimetric response between the species, several hypotheses could be considered: first, leaves of *M. sativa* and *O. europaea* correspond to non-sclerophyllous and sclerophyllous species, respectively, thus suggesting that different leaf tissue structure could be involved in this differential depolarizing response. Secondly, the chlorophyll-a and chlorophyll-b leaf content is different for each plant species^48–50^ so that the chlorotic symptoms development could be depending on the infected specimen chlorophyllic profile. Additionally, the type of pathogen which caused the chlorotic symptoms on both inspected leaves may also play a role in the differential depolarizing behavior: *M. sativa* was infected with the alfalfa mosaic virus (AMV) and *O. europaea* was infected with the fungus *V. oleaginea*. As far as the infection strategy of viruses is different from fungi, the chlorotic symptoms may manifest in different ways and lead to different physical transformations of plant tissues (thickness, stress resistance, turgor, structure, organization, etc.) of the unitary scatters (cells), therefore resulting in different depolarizing behavior. Moreover, Lanza et al.^51^ described the morphological changes induced by the infection of *V. oleaginea* on *O. europaea*, consisting of a progressive loss and degradation of plastids and chloroplasts in the palisade parenchyma cells. This degradation process leads to a release of cytoplasmic contents of palisade cells at advanced infection stages, which may affect the cuticle by reducing the resistance to water loss and causing stress on leaf tissues. Interestingly, the chlorotic ring on *O. europaea* might be also caused by fungal phytotoxins, as it was previously reported^52^. On the basis of the above comments, it is not surprising that a same symptom, i.e. chlorosis, is a consequence of different biological processes in different plants specimens, and therefore, may correspond to different polarimetric responses. In terms of the enhancement of the image contrast of disease symptoms, we have showed that differential information of polarimetric channels can well discriminate between different infection status (i.e., chlorosis, necrosis and healthy tissue). This has been demonstrated for *M. sativa* and *O. europaea*, and additionally confirmed in other plant specimens (see the complete list of analyzed specimens in Supplementary Table S1).

The pseudo-coloration is the second approach that we chose to better use the contrast enhancement provided by polarimetric images for a visual discrimination of features present in complex scattering media. As previously described, we performed a pseudo-coloration image processing based on triplets of polarimetric observables information codified in three color (R, G, B) channels. At this point we would like to emphasize that we use for the first time the pseudo-colored approach to the analysis of plant disease symptoms. Compared with previous references, the pseudo-coloration approach was improved by conducting two main modifications: (1) the depolarizing observables were not restricted to the IPPs space, but extended to an optimized selection of polarimetric observables within the IPPs and CPs spaces (2) an image filtering process, based on data obtained from a Boxplot analysis (see Fig. 5), was included in the process to largely discriminate between different tissue conditions (healthy/diseased).

In this section we bring the discussion to the pseudo-colored images resulting from the triplet *P*_2_, *P*_3_ and *P*_*S*_, as it is the most suitable one to construct the pseudo-colored functions for *M. sativa* and *O. europaea* samples (see Table 1). The pseudo-colored images obtained based on the IPPs lead to similar results, therefore, we invite the interested readers to consult results for IPPs in Supplementary Fig. S4. The final pseudo-colored images for *M. sativa* and *O. europaea*, presented in Fig. 6c and f, respectively, demonstrate a visual enhancement of disease symptoms: the extreme different coloration of the chlorotic lesions on *M. sativa* with respect to the healthy tissue of the leaf lamina (red and green regions on Fig. 6c) leads to a more accurate location of the diseased area. Similar behavior occurs for lesions on *O. europaea* leaf, where performed pseudo-colorations lead to a better delimitation of the different transitions from chlorotic spot to necrotic ring and healthy tissue of leaf lamina (Fig. 6f). Importantly, we remark here the fact that pseudo-colored images enhance the contrast between different tissue conditions, even more than the performance of isolated imaging of polarimetric observables for *M. sativa* and *O. europaea* samples (Fig. 6b,e, respectively). This behavior highlights the suitability of using this image-processing method for biological samples analysis. Particularly, the inspection and estimation of direct lesions, and the characterization and early detection of infection processes on plant tissues.

## Methods

### Sample description

The plant samples used in this work were a leaf of *Medicago sativa* specimen infected with *alfalfa mosaic virus* (AMV, which causes wilting or white flecks to necrotic wounds and chlorotic mosaics on leaves) and a leaf of *Olea europaea* specimen infected with *Venturia oleaginea* (causal agent of the olive leaf spot). This worldwide spread disease of olive may cause severe tree defoliation and a delay in fruit ripening, among other symptoms, thus leading to relevant yield losses.

Native from warmer-temperate climates of south-central Asia, *M. sativa* belongs to the Fabaceae family (legumes), it is cultivated worldwide for livestock feeding purposes. Despite the toxicity of unsprouted alfalfa, it is also suitable for human consumption in sprout stage or dehydrated. Regarding *O. europaea*, this species belongs to the Oleaceae family. Although the native species were found in eastern land regions around the Mediterranean Sea, his production is not limited to Mediterranean countries: *O. europaea* is cultivated in several countries such as South Africa, New Zealand, North and South America, and Australia. In addition to olive oil production and fruit consumption (olives), *O. europaea* trees are also grown for fine wood manufacturing.

T. Garnatje and J. Luque undertook the formal identification of the plant material used in this study. An herbarium voucher of both *M. sativa* and *O. europaea* are deposited in the Herbarium of the Botanical Institute of Barcelona (BC-983007 and BC-983006, respectively). All methods were performed in accordance with relevant guidelines and regulation.

### Mueller–Stokes formalism

Different mathematical approaches can be used to describe the polarimetric properties of material media^36^. Among those, in this work we use the Mueller–Stokes (M–S) formalism, because it is especially suited to deal with partially polarized or unpolarized light, as well as with depolarizing samples^53,54^. In this approach, polarization of light is described by means of Stokes vectors^36^ which are composed by four real coefficients., *S*_0_, *S*_1_, *S*_2_ and *S*_3_, whose physical interpretation is directly related to the irradiance (total intensity of the beam, *S*_0_) and the amount of light which is linearly polarized in vertical and horizontal (*S*_1_), 45° and 135° (*S*_2_) directions, and right or left-handed circularly polarized (*S*_3_). This Stokes formalism leads to a tridimensional representation of light states of polarization in the so-called Poincaré sphere^36^.

In the M–S formalism, polarimetric samples are described by 4 × 4 real coefficient matrices, the so-called Mueller matrices **M**, which can be understood as the polarimetric transfer functions of polarimetric systems. In addition, polarization of light exiting from a sample (represented by the ***S***_*out*_ Stokes vector) is linearly related with the input polarization (represented by the ***S***_*in*_ vector) through the Mueller matrix describing a sample. The Mueller matrix of a sample can be experimentally measured by using polarimeters^23,24^, and it encodes rich polarimetric information: dichroism (diattenuation and polarizance), retardance and depolarization. In the following, the polarimetric principle of a Mueller matrix experimental determination is briefly reviewed, but it is thoroughly described in Ref.^54^. By means of a complete image Mueller polarimeter, the sample is illuminated by a set of *n* different controlled light beam polarization states. Accordingly, the polarization of detected (imaged) light emerging from the sample is analyzed. To fully determine the experimental Mueller matrix, at least four input (and analyzed) states are required, which corresponds to 16 radiometric measurements. In the current work, a total of 36 measurements are taken: 6 input states of polarization (generators) and the corresponding 6 analyzers proposed in^55^, which are used for minimization of noise in radiometric measurements. The mathematical relationship between the set of incident states and the detected ones is given by the sample 4 × 4 Mueller matrix, **M**_S_, in the following way:

4 $${\mathbf{I}} = {\mathbf{S}}_{PSA} {\mathbf{M}}_{S} {\mathbf{S}}_{PSG} ,$$

where the detected radiometric measurements are given by the *n* × *n* matrix ***I***, ***S***_*PSG*_ is the 4 × *n* matrix of input set of polarized light beams where the *n* columns represent the different Stokes vectors used to illuminate the sample, and the *n* × 4 matrix ***S***_*PSA*_ which corresponds to the transposed input Stokes vectors. In this way, by computing the pseudoinverse of ***S***_*PSG*_ and ***S***_*PSA*_, matrices in Eq. (4), the corresponding Mueller matrix **M**_S_ of the sample can be directly deduced.

### Polarimetric observables

The complete set of polarimetric properties of the sample can be derived from the experimental Mueller matrix^36,53,54^. While some metrics (as dichroism) can be directly deduced from the Mueller matrix, other information as retardance and depolarization content are entangled in such a way that a straightforward interpretation is not possible and advanced algebraic methods of analysis, known as matrix decomposition methods are needed^56–60^. In this way, Mueller matrix **M** can be written as

5 $${\mathbf{M}} = m_{00} \left[ {\begin{array}{*{20}c} 1 & {{\mathbf{D}}^{T} } \\ {\mathbf{P}} & m \\ \end{array} } \right],$$

where *m*_00_ entails the non-polarized transmission/scattering of the sample, ***D*** and ***P*** are 3-dimensional vectors encoding diattenuation and polarizance, respectively, and 3 × 3 submatrix *m* entangles the retardance and depolarization in a complex way. Whereas ***D*** describes the dependency of intensity from emergent light from sample with the input state of polarization, ***P*** is related to the capability of said sample to polarize light when illuminated with an unpolarized state^36^. Regarding the *m*-submatrix, a commonly used formulism to gather entangled polarimetric properties is the so-called Lu-Chipman decomposition^55^, which describes any Mueller matrix **M** as the product of three 4 × 4 pure Mueller matrices (pure depolarizers, retarders and diattenuators) that synthesize well-defined polarimetric observables leading to an easier physical interpretation of the medium.

Regarding the depolarization behavior of media, a general quantitative indicator of the overall depolarizing power of the sample, the depolarization index *P*_*Δ*_, is commonly used^61,62^. Despite of the fact that *P*_*Δ*_ is suitable to represent homogeneous depolarization, it does not provide enough information regarding the situations where depolarization actually depends on the state of polarization of the illuminating beam. In this way, it is worth defining the covariance matrix **H** (associated with Mueller matrix, **M**)^61^:

6 $${\text{H}}({\text{M}}) = \frac{1}{4}\sum\limits_{i,j = 0}^{3} {m_{ij} \left( {\sigma_{i} \otimes \sigma_{j} } \right)} ,$$

where *m*_*ij*_ represent the Mueller matrix coefficients, σ are the Pauli matrices and $\otimes$ the Kronecker product. Since Mueller matrices are not Hermitian and thus we cannot ensure they are diagonalizable, we define the so-called indices of polarimetric purity (IPP)^33^ as a set of three real magnitudes, *P*_1_, *P*_2_ and *P*_3_, directly derived from the covariance matrix **H** eigenvalues:

7 $$P_{1} \equiv \frac{{\lambda_{0} - \lambda_{1} }}{{Tr{\mathbf{H}}}},\quad P_{2} \equiv \frac{{\lambda_{0} + \lambda_{1} - 2\lambda_{2} }}{{Tr{\mathbf{H}}}},\quad P_{3} \equiv \frac{{\lambda_{0} + \lambda_{1} + \lambda_{2} - 3\lambda_{3} }}{{Tr{\mathbf{H}}}},\quad \quad 0 \le P_{i} \le 1\quad (i = 1,2,3).$$

where *λ*-eigenvalues are taken in decreasing order as *λ*_0_ ≥ *λ*_1_ ≥ *λ*_2_ ≥ *λ*_3_ and IPP values are restricted to 0 ≤ *P*_1_ ≤ *P*_2_ ≤ *P*_3_ ≤ 1. Indices of polarimetric purity define a real tridimensional depolarization space whose interpretation, in addition to how much light is depolarized, is related with different depolarizing mechanisms in the sample. Therefore, the depolarization spaces can be potentially used to discriminate among structures which different depolarization signatures due to their properties and structure. Based on the idea of representing depolarization as the incoherent sum of four pure components^33^, IPPs correspond to the statistical weights of each component: *P*_1_ quantifies the relative portion of pure non-depolarizing component, *P*_2_–*P*_1_ the relative weight of a bidimensional depolarizer, *P*_3_–*P*_2_ the relative portion of an equiprobable mixture of three pure components (tridimensional depolarizer) and 1 − *P*_3_ is associated with an ideal depolarizer. In consequence, different IPPs values lead to the comprehension of the inherent depolarizing mechanisms of samples^34^. However, we can define depolarization index, *P*_*Δ*_, by means of IPPs but also eventually splitting depolarization information in the commonly used components of purity *P*,* D* and *P*_*S*_:

8 $$P_{\Delta } = \frac{1}{\sqrt 3 }\sqrt {2P_{1}^{2} + \frac{2}{3}P_{2}^{2} + \frac{1}{3}P_{3}^{2} } = \frac{1}{3}\sqrt {D^{2} + P^{2} + 3P_{S}^{2} } ,\quad \quad 0 \le P_{\Delta } \le 1,$$

where components of purity *P* and *D* are the polarizance and diattenuation vector magnitudes, respectively. The sphericity degree, *P*_*S*_, defines the contribution on depolarization which differs from dichroic origin. Therefore, depolarization index builds a common link between both purity spaces. Pure depolarizing systems are those which entail *P*_*Δ*_ = *P*_1_ = *P*_2_ = *P*_3_ = 0, meanwhile pure non-depolarizing media is characterized by *P*_*Δ*_ = *P*_1_ = *P*_2_ = *P*_3_ = 1. Recently, it has been demonstrated that the combined use of IPPs and components of purity is an ideal framework to describe depolarizing behavior of samples^37^.

### Complete image Mueller polarimeter

The polarimetric images shown in this work (Figs. 1, 2 and Supplementary Figs. S1 and S2) are gathered from the experimental Mueller matrices of the studied samples, which are acquired by means of a complete image Mueller polarimeter. By taking advantage of the wide spectral response of the light source, which actually covers the visible spectrum (from 400 to 700 nm approx.), we use three different illuminating wavelengths (625 nm, 530 nm and 470 nm) for the consequently inspection of the sample at different depths^47^. The polarimeter consists of two independent optical systems based on Parallel Aligned Liquid Crystals (PA-LC) retarders, mounted into two compact mobile arms respectively. The Polarization State Generator (PSG) optical design leads to generate any fully polarized state. It is composed by a linear polarizer oriented at 0° with respect to the laboratory vertical, followed by two PA-LC at 45° and 0°. Equivalently, the Polarization State Analyzer (PSA) is capable to detect any state of polarization. The PSA uses 6 optimized polarization analysis states^55^. In our instrument, both the PSG and the PSA consist of a linear polarizer followed by two Parallel-Aligned Liquid Crystal cells externally controlled by sending different voltages. The combination of both PSG and PSA, are used to record 36 intensity images which are used to deduce the Mueller matrix^55^. Regarding internal optical set-up, PSA has the same optical elements as PSG but arranged in reverse order. For the acquisition of sample intensity, a CCD camera is placed on the PSA system.

To perform the Mueller matrix measurements of biological samples, two main optical configurations are used. By tilting by 34° the PSG with respect to the horizontal laboratory reference and maintaining the PSA at 0° to avoid the ballistic reflection, we perform what we call scattering measurements. Complementary, by placing both PSG and PSA at 90° we perform transmission measurements. In both configurations, we selected from the whole sample, a region of interest (ROI) of 512 × 512 pixels, which corresponds to an area of 1.1 × 1.1 cm^2^. The detailed information about optical components and the visualization of measurement configurations (Figs. S5 and S6) is shown in Supplementary document.

## Supplementary Information

Supplementary Information.

## Data Availability

The datasets generated during and/or analysed during the current study are not publicly available due to the conduction of different research studies but are available from the corresponding author on reasonable request.
