# Supplementary material for: Polarimetric observables for the enhanced visualization of plant diseases
Source: Sci Rep. 2022 Aug 30;12:14743. doi: 10.1038/s41598-022-19088-6 (PMC9428171; doi:10.1038/s41598-022-19088-6)
Supplement: Supplementary file 1 — Supplementary Information. [file 41598_2022_19088_MOESM1_ESM.pdf]

Polarimetric observables for the enhanced visualization of plant diseases: supplementary document

Carla Rodríguez[1,*], Enrique Garcia-Caurel[2], Teresa Garnatje[3], Mireia Serra i Ribas[1], Jordi Luque[4], Juan Campos[1] and Angel Lizana[1]

[1]Optics group, Physics Department, Universitat Autònoma de Barcelona, Bellaterra, 08193, Spain
[2]LPICM, CNRS, Ecole Polytechnique, Institut Polytechnique de Paris, Palaiseau, 91120, France
[3]Botanical Institute of Barcelona (IBB, CSIC-Ajuntament de Barcelona), Barcelona, 08038, Spain
[4]Institute of Agrifood Research and Technology (IRTA), Cabrils, 08348, Spain
[*]carla.rodriguez@uab.cat

1. Plant material

In this work we have considered a set of 18 plant specimens and 73 inspected leaves in total. The plant samples were collected at different disease stages. To not to extent the content of the main manuscript, we limited the main discussion to the results related to two sample cases: a leaf of *M. sativa* specimen, showing chlorotic symptomatology due to the infection of alfalfa mosaic virus (AMV) and a leaf of *O. europaea* specimen, showing necrotic ring-like wounds and chlorotic halos due to the infection of *Venturia oleaginea*. In the following Table S1, we present the complete list of the 18 collected plant specimens, as well as their causal agent and short symptom description.

Table S1. Complete name list of the 18 collected specimens and their causal agent and symptom appearance. Different symptoms, which were not identified as chlorosis or necrosis were labeled as "other" discolorations observed on leaves. The selected specimens for this work are highlighted in gray.

Plant species	Pathogen – Organism group	Leaves inspected	Symptoms
Arauija sericifera	Undetermined organism	2	Chlorosis
Arbutus unedo	*Ruptoseptoria unedonis* – Fungi	1	Necrosis
Celtis australis	Undetermined virus	1	Other
	Undetermined phytoplasma	1	Chlorosis
Ficus carica	Undetermined organism	1	Other
	Fig Mosaic Virus – Virus	1	Chlorosis
Hedera helix	*Xanthomonas* – Bacteria	1	Other
	Mycosphaerella hederae-helicis – Fungi	1	Necrosis
	Undetermined organism	1	Chlorosis
Malva sylvestris	Malva Mosaic Virus – Virus	13	Chlorosis
	Undetermined bacterium	2	Necrosis
	Puccinia malvacearum – Fungi	20	Other
Medicago sativa	Alfalfa Mosaic Virus – Virus	1	Chlorosis
Morus sp.	Mulberry Mosaic Virus – Virus	1	Chlorosis
Olea europaea	*Venturia oleaginea* – Fungi	3	Necrosis
Parietaria officinalis	*Ramularia parietariae* – Fungi	1	Necrosis
Plantago coronopus	*Golovinomyces sordidus* – Fungi, powdery mildew	1	Other
Platanus × hybrida	*Erysiphe platani*– Fungi, powdery mildew	1	Other
	Corythucha ciliata – Insect	1	Chlorosis
Prunus dulcis	*Transzchelia discolor* – Fungi, rust	2	Other
	Panonychus ulmi – Arthropoda, mite	1	Chlorosis

	Polystigma amygdalinum – Fungi	1	Other
	Wilsonomyces carpophilus – Fungi	1	Necrosis
Quercus pubescens	Undetermined leaf miner – Arthropoda, insect	2	Other
	Erysiphe alphitoides – Fungi, powdery mildew	1	Other
Rubus idaeus	Undetermined organism	1	Necrosis
	Phragmidium violaceum – Fungi, rust	2	Other
Rumex pulcher	*Ramularia* sp. – Fungi	2	Other
Viburnum tinus	*Septoria* sp. – Fungi	1	Necrosis
	Undetermined fungus	1	Other
Vitis spp.	*Daktulosphaira vitifoliae* – Arthropoda, insect	1	Other
	Undetermined fungus	1	Other
	Uncinula necator – Fungi, powdery mildew	1	Chlorosis
	Guignardia bidlewii – Fungi	1	Necrosis

2. Qualitative analysis: polarimetric observables

In the following we present, as a complement to the material presented in the main manuscript, the non-polarized transmission / scattering images and the Mueller matrix-derived polarimetric observables (P_1, P_2, P_3, P_Δ, P, D and P_S) [1] for the leaf specimens of *Medicago sativa* and *Olea europaea* used in this study, both measured at 625 nm illumination wavelength.

Figure S1 shows a picture of the *M. sativa* specimen (Fig. S1a), the non-polarized transmission / scattering image (Fig. S1b) and the obtained polarimetric images of the Indices of polarimetric purity (P_1, P_2 and P_3), the depolarization index P_Δ, and the components of purity (P, D and P_S) (Figs. S1c to S1i, respectively), by means of the transmission-configuration measurement of the leaf. The depolarization inspection for the *M. sativa* sample clearly demonstrates an overall enhancement of image contrast and the revelation of the different spatial locations of the chlorotic wounds and the vascular structures (clearly seen in Fig. S1c) of the leaf, which are invisible in regular intensity images. Yellow arrows in Figs. S1d, S1e and S1f indicate the chlorotic tissue regions.

Regarding the *O. europaea* sample, Fig. S2 shows a picture of the specimen (Fig. S2a), the standard intensity image (Fig. S2b) and the obtained polarimetric images of the Indices of polarimetric purity (P_1, P_2 and P_3), the depolarization index P_Δ, and the components of purity (P, D and P_S) (Figs. S2c to S2i, respectively), by performing the scattering-configuration measurements of the beam part of the leaf. Inspected polarimetric channels demonstrate, in addition to the overall enhancement of image contrast, the accurate delimitation of the necrotic ring as well as the chlorotic spot (at the center of the lesion) and halo. Yellow and white arrows in Figs. S1d and S1e indicate the limits of the necrotic ring and the revealed diseased regions (chlorotic spot and halo) within the sample, respectively.

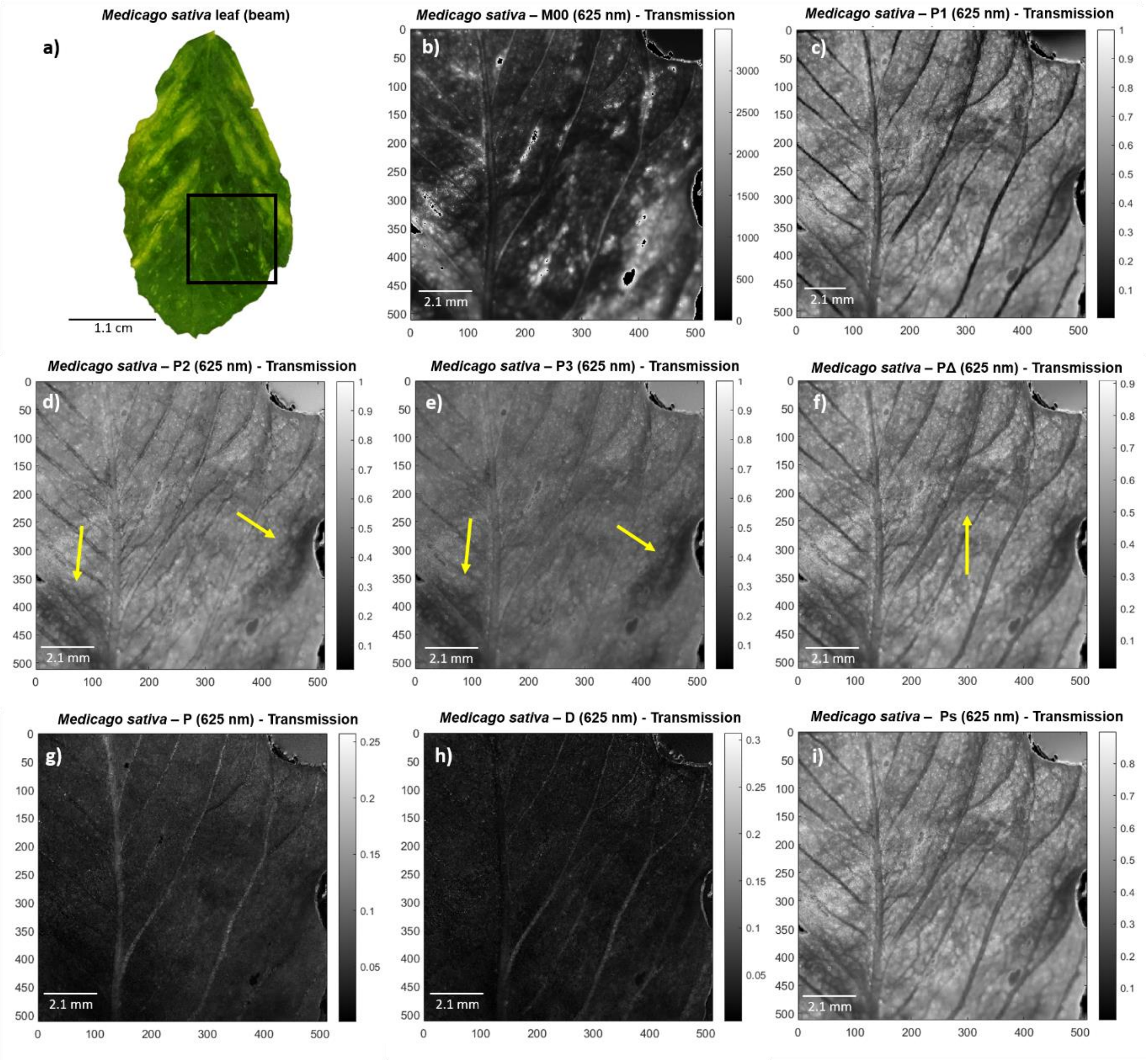

Figure S1. Images of polarimetric observables of *Medicago sativa* leaf. a) Picture of the beam part of the *M. sativa* leaf used in this study. Black square denotes for selected region of interest (ROI) analyzed in remaining images, b) regular intensity image (M_{00}) of the *M. sativa* transmission ROI and its corresponding polarimetric observables c) P_1, d) P_2, e) P_3, f) P_Δ, g) P, h) D and i) P_S for visual comparison. All images correspond to 625 nm illumination wavelength measurements performed at transmission set-up configuration. Yellow arrows indicate the locations of chlorotic tissue regions within the sample.

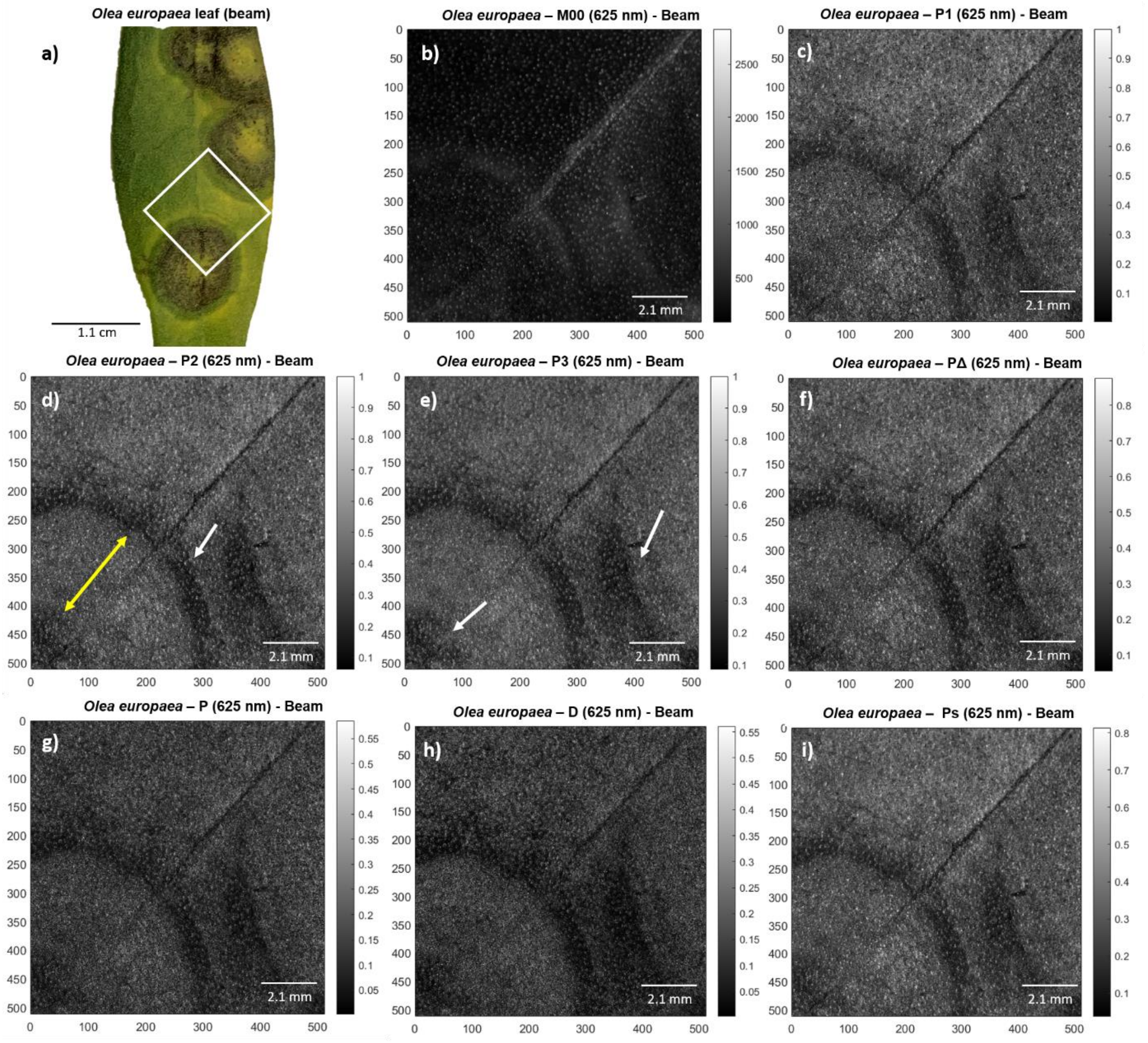

Figure S2. Images of polarimetric observables of *Olea europaea* leaf. a) Picture of the beam part of the *O. europaea* leaf used in this study. White square denotes for selected region of interest (ROI) analyzed in remaining images, b) regular intensity image (M_{00}) of the *O. europaea* beam ROI and its corresponding polarimetric observables c) P_1, d) P_2, e) P_3, f) P_Δ, g) P, h) D and i) P_S for visual comparison. All images correspond to 625 nm illumination wavelength measurements performed at scattering set-up configuration. Yellow and white arrows indicate the locations of necrotic ring-like and other injured tissue regions (chlorotic spot and halo) within the sample.

3. Exploratory analysis of polarimetric observables data

This section is devoted to demonstrate the reliability of the method used for the selection of the polarimetric observables triplet. As described in the main manuscript, our purpose is to achieve a pseudo-colored image which provides significant results in terms of disease symptom visualization, i.e., to enhance visual differentiation between healthy and diseased tissue regions of inspected plants. To this aim, we decided to base the selection of the observable triplet in terms of observable median values leading to largest polarimetric differences between healthy and wounded regions of inspected specimen leaves. Under this scenario, it is important to know how our data distributions behave and if we are dealing with reliable median values. To do so, we performed the Boxplot analysis [2,3] (Fig. 5 of the main manuscript) for the selected purity spaces (P_1,

P_2, P_3, P, D and P_S) regarding different tissue conditions regions (healthy, chlorotic and necrotic, shown in Fig. 4 of the main manuscript) of *M. sativa* and *O. europaea* specimens. In this context, we studied the percentage of outliers as well as the standard errors of the medians for each observable data distribution.

3.1 Outliers inspection

Regarding Boxplot charts (Fig. 5 of the main manuscript), the polarimetric data distributions for each type of tissue condition (healthy, chlorotic and necrotic) present a certain amount of outlier values, which are known as particular observation data points which lie at an abnormal distance (1.5 times –mild– or 3 times –extreme– the distance between first and third quartile) from the first or third quartiles of the distribution (illustrated as circles –mild– and stars –extreme–, respectively, in Fig. 5 of the main manuscript).

The calculated outlier percentages for each polarimetric data observable and for each tissue condition studied are provided in Table S2. Importantly, note that a low outlier percentage from a polarimetric observable distribution within a given tissue condition indicates that only few data points differ, in an abnormal way, from the whole tendency. Consequently, we can demonstrate the homogeneity selection of the different tissue regions (healthy, chlorotic and necrotic). In particular, the minimum percentage of outliers (0.44%) is demonstrated for the spherical degree, P_S, distribution within the healthy region of the *O. europaea* leaf, whereas the largest (3.67%) lies in the distribution of the index of polarimetric purity P_1 for the chlorotic tissue region of the *M. sativa* inspected leaf. From data in Table S2 we can ensure the validity of most polarimetric data used for the study presented in this manuscript.

Table S2. Polarimetric observables outliers in percentage (%) for healthy and diseased regions in *Medicago sativa* and *Olea europaea* inspected leaves. Maximum and minimum outlier % values are highlighted in gray.

			P_1	P_2	P_3	P	D	P_S
Medicago sativa	**% Outliers**	**Healthy**	1.92%	1.39%	1.21%	1.32%	1.21%	1.32%
		**Chlorosis**	3.67%	1.50%	2.00%	1.16%	2.08%	3.08%
Olea europaea	**% Outliers**	**Healthy**	0.58%	1.03%	0.97%	0.77%	0.58%	0.44%
		**Chlorosis**	0.82%	0.89%	1.21%	0.57%	1.03%	0.93%
		**Necrosis**	1.24%	1.03%	1.63%	0.90%	1.39%	1.30%

3.2 Analysis on median values

Once the validity of the selected tissue regions of both inspected samples is demonstrated, we can ensure the correct treatment of polarimetric data as well as its interpretation in terms of discriminative potential. Regarding the last, we visually deduce from median values extended red-dotted lines in Fig. 5 of the main manuscript, the potential of several polarimetric observables to set apart the different type of tissues (healthy, chlorotic and necrotic) within a sample. This is because in the Boxplot analysis, if the median of one tissue does not coincide within the box of another tissue for a given polarimetric observable, those tissues can be interpreted as statistically discriminable within such observable (i.e., the median value of one class, falls out the 75% of data corresponding to the other class).

For the correct quantification of the visual discrimination for both *M. sativa* and *O. europaea* samples, the calculated median values and the corresponding associated errors for each polarimetric observables (P_1, P_2, P_3, P, D and P_S) and type of tissue (healthy, chlorotic and necrotic) are presented in Table S3. In this context, the median values for the studied polarimetric observables present, overall, low standard deviations of the median. This behavior is correlated with reliable polarimetric median values, allowing us to perform comparatives between the discriminative potential of the different metrics (P_1, P_2, P_3, P, D and P_S) through bloxplot analysis.

Table S3. Polarimetric observables median values and standard deviations on healthy, chlorotic and necrotic regions of the studied leaves of *Medicago sativa* and *Olea europaea*.

		P_1	P_2	P_3	P	D	P_S
Medicago sativa	**Healthy**	0.187 ± 0.034	0.243 ± 0.041	0.498 ± 0.047	0.117 ± 0.025	0.102 ± 0.022	0.236 ± 0.031
	**Chlorosis**	0.278 ± 0.077	0.363 ± 0.095	0.612 ± 0.094	0.169 ± 0.048	0.144 ± 0.036	0.325 ± 0.074
Olea europaea	**Healthy**	0.033 ± 0.013	0.068 ± 0.018	0.012 ± 0.027	0.023 ± 0.010	0.024 ± 0.010	0.055 ± 0.014
	**Chlorosis**	0.012 ± 0.004	0.029 ± 0.007	0.057 ± 0.010	0.022 ± 0.007	0.019 ± 0.004	0.018 ± 0.004
	**Necrosis**	0.087 ± 0.027	0.149 ± 0.036	0.238 ± 0.052	0.036 ± 0.016	0.035 ± 0.016	0.125 ± 0.028

3.3 Pseudo-coloration functions implementation

In this section we present the detailed description of the performed steps regarding the pseudo-coloration of *M. sativa* and *O. europaea* inspected samples, they being based on two triplets of polarimetric observables, (P_2, P_3, P_S) and (P_1, P_2, P_3).

The main goal is to achieve an image visual enhancement of different plant tissue conditions (healthy and diseased regions) when compared with non-pseudocolored polarimetric images. To construct the pseudo-colored images, as explained in the main text, we need to define some threshold values discriminating between tissues conditions (chlorosis, healthy and necrosis), which were derived from Boxplot analysis (see Fig. 5 in main manuscript). In the following, we explain in detail the method to set the different thresholds, corresponding to each polarimetric observable contained into a given triplet.

Note that the different tissue conditions are well differentiated when represented within the boxplot charts (see Fig. 5 in main manuscript). Taking adventatge of this situation, we can set a threshold able to discriminate between each pair of tissue conditions in a binary way. In the case of the *M. sativa* sample, we only need to discriminate between chlorotic and healthy tissues, so only one threshold value is required. In turn, in the case of the *O. europaea* sample, we need to discriminate between healthy, chlorotic and nectrotic tissues, so two thresholds are required: (1) chlorotic-healthy and (2) necrotic-healthy thresholds (note that healthy tissues gets intermediate values between chlorotic and nectrotic tissues, so no more threshods are needed – see Fig. 4 in main manuscript). In addition, this threshold assignment must be repeated for each polarimetric observable present in one of the studied triplets (P_1, P_2, P_3, and P_S). Under this scenario, for a particular observable and for a particular case between two tissue conditions to be discriminated, let us call them condtion A and B, we calculated, from the Boxplot chart, the observable value of the first quartile (bottom side of the box), for the tissue condition with the highest mean value between A and B, as well as the observable value of the third quartile (upper side of the box), for the tissue condition with the smallest mean between A and B. Finally, the threshold value is directly obtained by conducting the difference, in each case, between these two quartile values (third and first). Obtained quartile values for the different polarimetric observables (columns) and for the different tissue conditions (rows) present in the two studied samples (*M. sativa* and *O. europea*) are provided in Table S4. Resulting thresholds are presented in following Table S5.

Table S4. First and third quartiles for chlorotic, healthy and necrotic tissue distributions for each polarimetric observable of *Medicago sativa* and *Olea europaea* leaf studied samples.

		P_1		P_2		P_3		P_S	
		Q_1	Q_3	Q_1	Q_3	Q_1	Q_3	Q_1	Q_3
Medicago sativa	**Healthy**	-	0.209	-	0.271	-	0.531	-	0.271
	**Chlorotic**	0.232	-	0.303	-	0.552	-	0.303	-
Olea europaea	**Chlorotic**	-	0.015	-	0.034	-	0.064	-	0.022
	**Healthy**	0.024	0.043	0.056	0.082	0.104	0.141	0.046	0.066
	**Necrotic**	0.069	-	0.126	-	0.207	-	0.108	-

Table S5. Definition of leaf lesion types by means of threshold pixel values for chlorotic, healthy and necrotic tissues for each polarimetric observable of *Medicago sativa* and *Olea europaea* leaf studied samples.

		**Threshold: P_1**	**Threshold: P_2**	**Threshold: P_3**	**Threshold: P_S**
Medicago sativa	**Healthy**	$P_1 < 0.220$	$P_2 < 0.287$	$P_3 < 0.542$	$P_S < 0.269$
	**Chlorotic**	$P_1 \geq 0.220$	$P_2 \geq 0.287$	$P_3 \geq 0.542$	$P_S \geq 0.269$
Olea europaea	**Chlorotic**	$P_1 \leq 0.020$	$P_2 \leq 0.045$	$P_3 \leq 0.084$	$P_S \leq 0.034$
	**Healthy**	$0.056 > P_1 > 0.020$	$0 .104 > P_2 > 0.045$	$0.174 > P_3 > 0.084$	$0.087 > P_S > 0.034$
	**Necrotic**	$P_1 \geq 0.056$	$P_2 \geq 0.104$	$P_3 \geq 0.174$	$P_S \geq 0.087$

Note that in a boxplot representation, the box sides (upper and bottom) define the first and third quartiles respectively, and thus the 50% of data corresponding to a given distribution falls into the box. Therefore, above the first quartile, as well as under the third quartile, there is represented the 75% of the data related to a given data distribution. Importantly, the thresholds constructed by using the above-stated approach, are set halfway between the first and third quartiles of the particular pair of tissues being discriminated, and thus, this construction ensures a tissue condition recognition in all the cases superior to the 75% in each polarimetric channel studied. This is always true if the boxes corresponding to the different tissues to be discriminated are not overlapped in a particular polarimetric observable (as it is the case of the observables selected for the triplets, see Fig. 5 of the main manuscript). In fact, this recognition rate should be even larger if taking into account that the the remaining data (25% of data, which is represented in the whiskers) of each tissue is generally not linearly distributed throught the whiskers but especially close to the box sides (i.e., certain data between boxes above/below the threshold is still well recognized). For the sake of clarity, a representative graphic example of quartiles selection and threshold location is shown in Fig. S3.

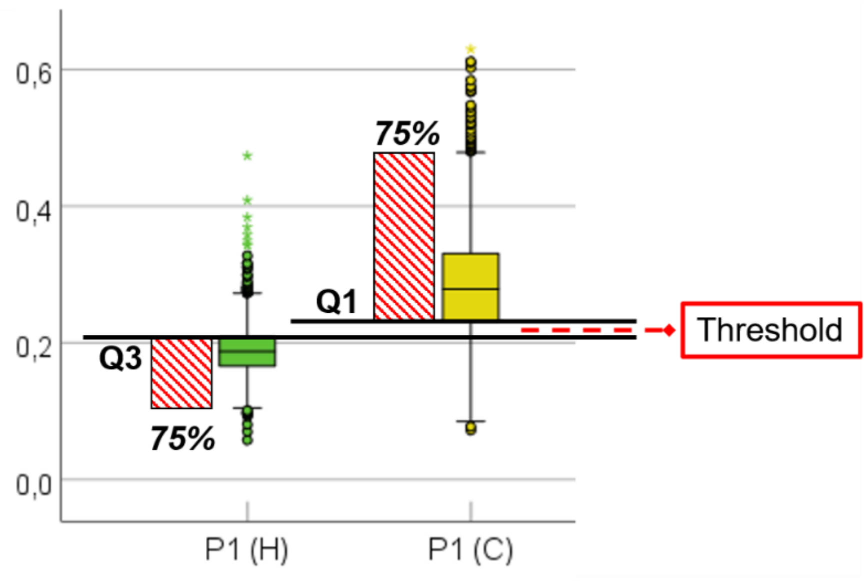

Figure S3. Representative example of quartile values location and threshold calculation for chlorotic and healthy P_1 distributions of the *Medicago sativa* inspected sample. The relative position of first and third quartiles (labels Q1 and Q3, respectively) for healthy (H) and chlorotic (C) distributions, are indicated by means of continuous black lines, whereas the location of the corresponding threshold (mean value between Q3 and Q1) is red-lined. Red-dashed boxes illustrate the percentage of data explained under the third quartile and above the first of the healthy and chlorotic polarimetric observable P_1 data distribution.

Once the thresholds were obtained as previously discussed (see values in Table S5), they were used to implement the pseudo-colored images. To do so, we associated each one of the three primary colors (R, G, B) to the different tissue conditions of the inspected samples: chlorotic regions were associated to red, healthy tissues to green and necrotic ones to blue. Afterwards, a binary colored image (color/black) is constructed for each tissue condition: values above/below the corresponding threshold are assigned to the particular tissue condition color (R-chlorotic, G-healthy, B-necrotic) or to black, respectively. Consequently, for each particular polarimetric observable P_i ($i=1, 2, 3, S$), we construct three independent images, one per primary color (R, G and B), each one carrying the information corresponding to a given tissue condition. Afterwards, the three binary colored-images corresponding to a particular polarimetric observable P_i, are added by means of a linear combination, leading to a first pseudo-colored image, as showin in Eq. (S1):

$$P_{i,approach}(x,y) = P_{i,Red-Chlorotic}(x,y) + P_{i,Green-Healthy}(x,y) + P_{i,Blue-Necrotic}(x,y). \qquad \text{(S1)}$$

Therefore, according to Eq. (S1), the first-approach pseudo-colored functions for the different polarimetric observables in the case of *M. sativa* are defined as:

$$\begin{aligned} P_{1,approach,MS}(x,y) &= P_{1,R-Chlorotic}(x,y) + P_{1,G-Healthy}(x,y), \\ P_{2,approach,MS}(x,y) &= P_{2,R-Chlorotic}(x,y) + P_{2,G-Healthy}(x,y), \\ P_{3,approach,MS}(x,y) &= P_{3,R-Chlorotic}(x,y) + P_{3,G-Healthy}(x,y), \\ P_{S,approach,MS}(x,y) &= P_{S,R-Chlorotic}(x,y) + P_{S,G-Healthy}(x,y), \end{aligned} \qquad \text{(S2)}$$

and for the case of *O. europaea*:

$$
\begin{aligned}
&P_{1,approach,OE}(x,y) = P_{1,\mathrm{R}-Chlorotic}(x,y) + P_{1,G-Healthy}(x,y) + P_{1,B-Necrotic}(x,y),\\
&P_{2,approach,OE}(x,y) = P_{2,R-Chlorotic}(x,y) + P_{2,G-Healthy}(x,y) + P_{2,B-Necrotic}(x,y),\\
&P_{3,approach,OE}(x,y) = P_{3,R-Chlorotic}(x,y) + P_{3,G-Healthy}(x,y) + P_{3,B-Necrotic}(x,y),\\
&P_{S,approach,OE}(x,y) = P_{S,R-Chlorotic}(x,y) + P_{S,G-Healthy}(x,y) + P_{S,B-Necrotic}(x,y).
\end{aligned} \tag{S3}
$$

where *MS* and *OE* sub-labels denote for *M. sativa* and *O. europaea*, respectively, and *R, G* and *B* for red, green and blue primary colors associated to the chlorotic, healthy and necrotic tissue regions, respectively. Note that the term regarding to necrotic tissues in *M. sativa* (Eq. (S2)) is equal to zero ($P_{i,\ Blue\text{-}Necrotic}$ = 0) because there is no necrotic content within the inspected sample. Afterwards, and recalling the selected polarimetric triplets (i.e., (1) the Components of Purity: P_2, P_3, P_S and; (2) the IPPs: P_1, P_2, P_3), each of the RGB-layered images obtained from Eqs. (S2) and (S3) is put together by means of a second linear combination. In this way, the particular information of each polarimetric observable into a triplet is combined in a final RGB-layered pseudo-colored image. The final image linear combinations for the triplets (P_2, P_3, P_S) and (P_1, P_2, P_3) for *M. sativa* and *O. europaea* inspected samples are presented in the following equations (S4) and (S5), respectively:

$$
\begin{aligned}
&Pseudo\#1(x,y)_{MS} = P_{2,approach,MS}(x,y) + P_{3,approach,MS}(x,y) + P_{S,approach,MS}(x,y),\\
&Pseudo\#2(x,y)_{MS} = P_{1,approach,MS}(x,y) + P_{2,approach,MS}(x,y) + P_{3,approach,MS}(x,y),
\end{aligned} \tag{S4}
$$

$$
\begin{aligned}
&Pseudo\#1(x,y)_{OE} = 2\times[P_{2,approach,OE}(x,y) + P_{3,approach,OE}(x,y) + P_{S,approach,OE}(x,y)],\\
&Pseudo\#2(x,y)_{OE} = 2\times[P_{1,approach,OE}(x,y) + P_{2,approach,OE}(x,y) + P_{3,approach,OE}(x,y)],
\end{aligned} \tag{S5}
$$

where *MS* and *OE* sub-labels denote for *M. sativa* and *O. europaea*. For simplicity, we chose the weigths for polarimetric triplets to be the unit. Nevertheless, the overall low values on polarimetric observables on *O. europaea* lead to a dark tone final image. To improve the visualization, the weights were pondered by a factor 2.

For visual comparison, in Fig. S4 we present the regular intensity images, the images for the indices of polarimetric purity P_2 and P_1, and the obtained pseudo-colored images by means of performing the above-presented relationships in Eqs. (S4) and (S5) for *M. sativa* and *O. europaea*. The final pseudo-coloration clearly demonstrates, as initially stated, the image visual enhancement between pathology symptoms (injured regions) and healthy tissue within the inspected samples. A detailed discussion of the obtained results is provided in the discussion section of the main manuscript.

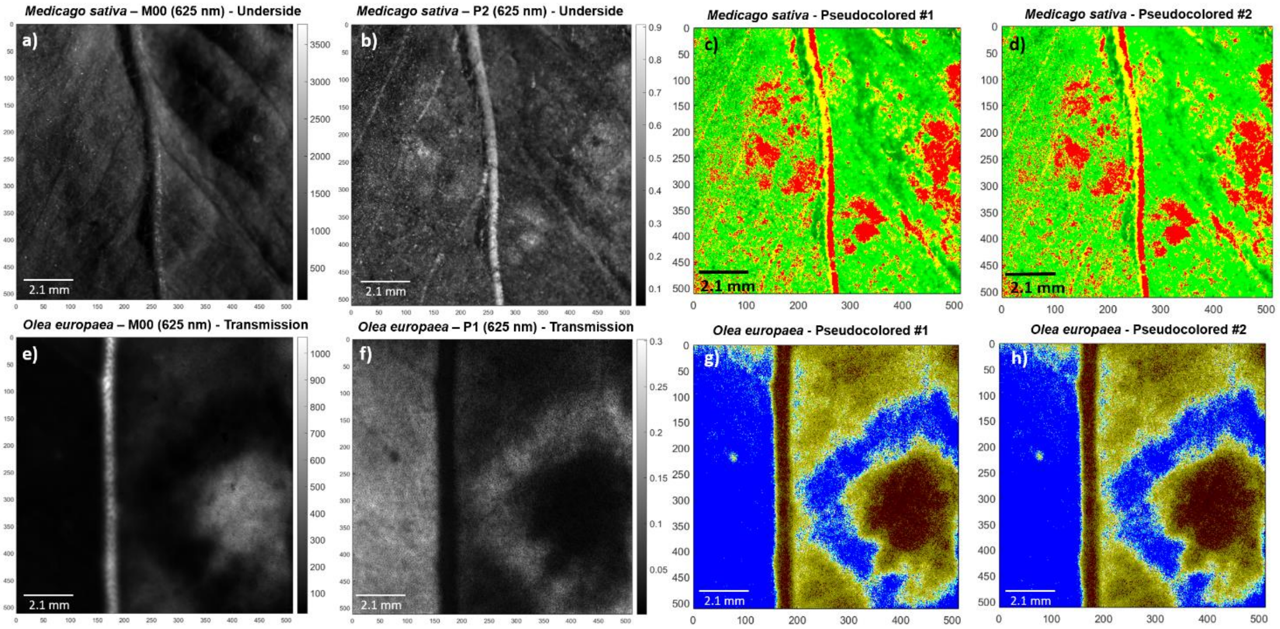

Figure S4. Visual comparison of *Medicago sativa* leaf (top row): a) 625 nm intensity image (M_{00}), b) polarimetric purity index P_2, c) processed image by means of #1 pseudo-coloration and d) processed image by means of #2 pseudo-coloration; Visual comparison of *Olea europaea* leaf (bottom row): e) 625 nm intensity image (M_{00}), f) polarimetric purity index P_1, g) processed image by means of #1 pseudo-coloration and h) processed image by means of #2 pseudo-coloration.

4. Complete image Mueller polarimeter description

The experimental Mueller matrices of the collected samples were acquired by means of a complete image Mueller polarimeter working at three different illumination wavelengths (625 nm, 530 nm and 470 nm), covering the visible range, and two measurement configurations: scattering and transmission. Importantly, the polarimeter used in this work consists of two independent optical systems gathered into two mobile compact arms, the polarization state generator (PSG) and the polarization state analyzer (PSA). The optical design for PSG consists on the arrangement of a linear polarizer oriented at 0º with respect to the laboratory vertical, followed by two Parallel Aligned Liquid Crystal (PA-LC) retarders at 45º and 0º, respectively. PSA optical set-up has the same elements as PSG but placed in the inverse order, but additionally placing a CCD camera for the capture of the sample intensity. This architecture leads to the capability of generating and analyzing, respectively for the PSG and PSA systems, any fully polarized state. Remark that scattering measurements are performed by illuminating the sample by placing the PSG at 34º with respect to the laboratory horizontal and the PSA in vertical position to avoid the direct reflections and collect scattered light. Transmission configuration is characterized by placing both PSG and PSA at 0º with respect to laboratory horizontal. Visual representation of both optical set-up configurations as well as the inner components is shown in Figs. S5 and S6, respectively.

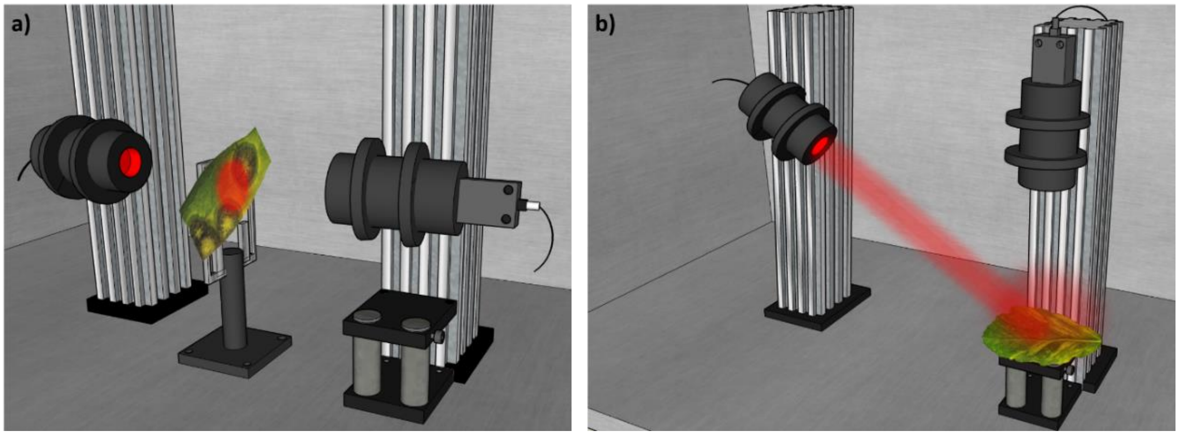

Figure S5. 3D representation of the complete image Mueller polarimeter used in this study at a) transmission configuration and b) scattering configuration.

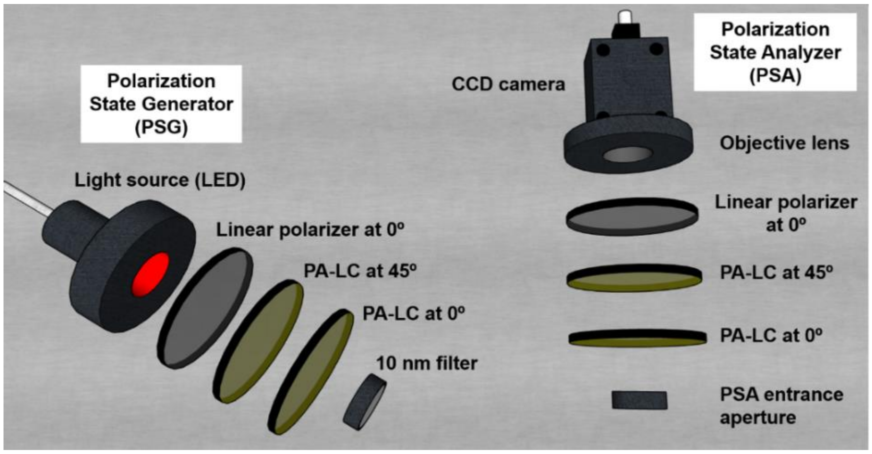

Figure S6. 3D representation of the polarization state generator (PSG) and polarization state analyzer (PSA) optical components arrangement.

In the following we provide the detailed information about the optical components within the polarimeter: the illumination is performed by means of we use a four-wavelength high-power Thorlabs LED source (LED4D211, operated by DC4104 drivers distributed by Thorlabs) complemented with 10 nm dielectric bandwidth filters distributed by Thorlabs: FB530-10 and FB470-10 for green and blue wavelengths, respectively. Imaging is performed by means of a 35 mm focal length Edmund Optics TECHSPEC® high resolution objective followed by an Allied Vision Manta G-504B CCD camera, with 5 Megapixel GigE Vision and Sony ICX655 CCD sensor, 2452(H) × 2056(V) resolution and cell size of 3.45 µm × 3.45 µm, so a spatial resolution of 22 µm is achieved. Regarding the two different linear polarizers: the one arranged on PSG is a Glan-Thompson prism-based CASIX whereas the placed in PSA is a dichroic sheet polarizer distributed by Meadowlark Optics. The four Parallel Aligned Liquid Crystals are Variable Retarders with Temperature Control (LVR-200-400-700-1LTSC distributed by Meadowlark Optics).

5. References

1. Gil, J. J. & Ossikovski, R. *Polarized Light and the Mueller Matrix Approach* (CRC Press, Boca Raton, 2016).

2. Tukey, J. W. *Exploratory Data Analysis* (Addison-Wesley, 1977).

3. McGill, R., Tuckey, J. W. & Larsen, W. A. Variations of Box Plots, *The Am. Stat*. **32**(1), 12-16; 10.2307/2683468 (1978).
